# Effects of Intermittent Hypoxia in Training Regimes and in Obstructive Sleep Apnea on Aging Biomarkers and Age-Related Diseases: A Systematic Review

**DOI:** 10.3389/fnagi.2022.878278

**Published:** 2022-05-23

**Authors:** Belay Tessema, Ulrich Sack, Brigitte König, Zoya Serebrovska, Egor Egorov

**Affiliations:** ^1^Institute of Clinical Immunology, Faculty of Medicine, Leipzig University, Leipzig, Germany; ^2^Institute of Medical Microbiology and Epidemiology of Infectious Diseases, Faculty of Medicine, Leipzig University, Leipzig, Germany; ^3^Department of Medical Microbiology, College of Medicine and Health Sciences, University of Gondar, Gondar, Ethiopia; ^4^Department of General and Molecular Pathophysiology, Bogomoletz Institute of Physiology, National Academy of Sciences of Ukraine, Kyiv, Ukraine; ^5^IPAM Institute for Preventive and Anti-Aging Medicine, Berlin, Germany

**Keywords:** IHNT, IHHT, OSA, aging biomarkers, age-related diseases

## Abstract

Several studies have assessed the effects of intermittent hypoxia-normoxia training (IHNT), intermittent hypoxia-hyperoxia training (IHHT), and obstructive sleep apnea (OSA) on aging and age-related diseases in humans; however, the results remain contradictory. Therefore, this review aims to systematically summarize the available studies on the effects of IHNT, IHHT, and OSA on aging and age-related diseases. Relevant studies were searched from PubMed, Google Scholar, Cochrane Library databases, and through manual searching from reference lists of eligible studies. A total of 38 eligible studies were included in this systematic review. IHHT and IHNT provide positive effects on several age-related parameters including quality of life, cognitive and physical functions, plasma level of glucose and cholesterol/LDL, systolic blood pressure, red blood cells, and inflammation. Moreover, moderate intermittent hypoxia induces telomerase reverse transcriptase (TERT) activity and telomere stabilization, delays induction of senescence-associated markers expression and senescence-associated β-galactosidase, upregulates pluripotent marker (Oct4), activates a metabolic shift, and raises resistance to pro-apoptotic stimuli. On the contrary, intermittent hypoxia in OSA causes hypertension, metabolic syndrome, vascular function impairment, quality of life and cognitive scores reduction, advanced brain aging, increase in insulin resistance, plasma hydrogen peroxide, GSH, IL-6, hsCRP, leptin, and leukocyte telomere shortening. Thus, it can be speculated that the main factor that determines the direction of the intermittent hypoxia action is the intensity and duration of exposure. There is no direct study to prove that IHNT/IHHT actually increases life expectancy in humans. Therefore, further study is needed to investigate the actual effect of IHNT/IHHT on aging in humans.

**Systematic Review Registration:**

www.crd.york.ac.uk/prospero, identifier CRD42022298499.

## Introduction

Eukaryotic cells require sufficient concentration of oxygen (O_2_) to ensure survival and maintain a variety of biological activities. Due to the important roles of O_2_ in metabolism, respiration, and survival, eukaryotes have developed an effective and rapid O_2_ sensing system (Taylor, 2008). When the O_2_ demand surpasses its supply, O_2_ levels in the local tissues or whole body decrease (called hypoxia), leading to threatening physiological functions, metabolic crisis, and viability. These hypoxic responses are controlled by hypoxia-inducible factors (HIFs) (Semenza, 2000). The HIF-pathways cross talk with the AMP-activated protein kinase (AMPK), mammalian target of rapamycin complex 1 (mTORC1), nuclear factor κB (NFκB), sirtuins, and mechanistic UNC-51-like kinase 1 (ULK1) pathways in hypoxia and aging (Ruderman et al., 2010; Hong et al., 2014; Antikainen et al., 2017; Pan and Finkel, 2017).

Exposures to acute prolonged (4 h) severe hypoxia and long-term hypoxia increase oxidative stress (Joanny et al., 2001; Magalhães et al., 2004). Both duration and intensity of hypoxic exposure are known to play an important role in causing oxidative stress. It is known that a moderate amount of reactive oxygen species (ROS) is important to physiological processes leading to positive cellular adaptive responses, while large amounts of ROS can damage proteins, lipids, and DNA and result in pathophysiological responses (Hancock et al., 2001; Rains and Jain, 2011). Therefore, exposing healthy people or chronic patients to severe hypoxia could be harmful, generate high levels of ROS, and facilitate the progression of illness. On the contrary, a training program consisting of 15–24 sessions of intermittent hypoxia (10–12% O_2_)–normoxia (21% O_2_) (IHNT) and intermittent hypoxia (10–12% O_2_)–hyperoxia (30–40% O_2_) (IHHT) has been considered as an effective treatment in patients with a variety of age-related diseases (Burtscher et al., 2004; Bayer et al., 2017; Glazachev et al., 2017; Dudnik et al., 2018).

One of the key activating mechanisms for adaptive responses to intermittent hypoxia training (IHT) is the induction of ROS, which is involved in cell signaling during cell growth or tissue repair. Moreover, exposure to ROS induces the expression of certain genes, the products of which promote defense against cellular stress (Sen and Packer, 1996; Debevec et al., 2017). This effect is associated with an increase in antioxidant capacity, both enzymatic and non-enzymatic (Zepeda et al., 2013; Heß et al., 2019). The main pathways in adaptive responses are considered to be reductive stress within the mitochondria, decreased mitochondria redox potential, xanthine oxidase pathway activation, and augmented catecholamine production (Lukyanova et al., 2009; Lukyanova and Kirova, 2011). Mild hypoxic episodes exposures for short time can provide protection to specific organs, tissues, or cells against more severe hypoxia and ischemia (Debevec et al., 2015) and provide a cost-effective strategy for improving metabolic function (Mackenzie and Watt, 2016). According to some reports, IHHT enhances the ROS-induced signal without increasing hypoxia. This leads to the significant synthesis of protective intracellular protein molecules, mainly with antioxidant function (antioxidant enzymes, iron-binding proteins, and heat shock proteins) (Arkhipenko et al., 2005; Sazontova et al., 2017). The IHT also influences several underlying mechanisms of aging, such as the expression of p53 and p66 proteins that modulate inflammation and apoptosis, and tissue repair and DNA maintenance (Bianchi et al., 2006).

Human telomeres comprise a series of TTAGGG tandem repeats at the end of chromosomes, which maintains chromosomal integrity and stability and allows effective replication of DNA. Telomerase is responsible for maintaining telomere length (TL) by synthesizing TTAGGG repeats at the end (Blackburn, 2001). Some base pairs are normally lost from the terminal region of chromosomes during each cell division (Prasad et al., 2017). Therefore, TL has been used as a common biomarker of aging. In animal models, shortened or lengthened telomeres mean decreased or increased lifespan of mice, respectively (Armanios et al., 2009). Shortened telomeres may lead to the cessation of cell division, thus promoting cellular senescence and apoptosis (Prasad et al., 2017). Meanwhile, shortened telomeres are more sensitive to systematic inflammation and oxidative stress, which can further promote aging (Masi et al., 2011).

On the contrary, the mechanisms of maladaptive responses to intermittent hypoxia are mostly associated with OSA (Dempsey and Morgan, 2015; Beaudin et al., 2017). OSA is a common chronic sleep-related breathing disorder characterized by recurrent pauses in breathing, which are caused by collapsing upper airways (Lévy et al., 2015). The prevalence of OSA in the general population reaches up to 23% in women and 50% in men, and the risk of the disorder increases with higher body mass index, advancing age, and male sex (Mokros et al., 2018). OSA is commonly associated with age-related disorders such as hypertension, cardiovascular disorders, metabolic abnormalities like obesity or type 2 diabetes mellitus (Bradley and Floras, 2009; Gabryelska et al., 2021), and cancer (Hunyor and Cook, 2018). OSA causes chronic intermittent hypoxia, which is the main pathophysiological mechanism causing chronic inflammation and oxidative stress (Lévy et al., 2015), thereby leading to cellular damage and accelerated aging (López-Otín et al., 2013). Some studies showed that OSA is significantly related to shortened TL (Fyhrquist and Saijonmaa, 2012); however, Kim et al. (2010) and Polonis et al. (2017) demonstrated that TL elongated in moderate-to-severe OSA.

Several studies have reported the effects of IHNT, IHHT, and OSA on aging and age-related diseases in humans and cell culture; however, some of the findings are contradictory. Moreover, it is not well documented whether hypoxic conditioning *via* IHNT or IHHT may induce different physiological responses and whether one method may provide more advantages over the other. Furthermore, less is known whether acutely exposing people to severe IHNT and IHHT is associated with a potentially harmful change in their redox status. Therefore, this review aims to systematically summarize the available literature on the effects of IHNT, IHHT, and OSA on aging and age-related diseases.

## Methods

### Protocol Development and Review Approach

This systematic review was done based on the population, intervention, control, and outcomes (PICO) questions for interval hypoxia studies and condition-context-population (CoCoPop) review method for sleep apnea studies. PICO questions include the following: P = patients of all age groups with age-related diseases, I = IHNT and IHHT; C = patients with normoxia (21% O_2_) exposure; O = changes of age-related diseases condition and/or aging markers; study design = randomized controlled clinical trial, non-randomized controlled clinical trials, or non-controlled randomized clinical trial study. CoCoPop review questions include the following: Co = sleep apnea and age-related diseases, Co = sleep apnea occurs at night time in a supine position and in a state of sleep accompanied by hypercapnia, age-related diseases diagnosed by disease-specific tests, and aging measured based on aging biomarkers including telomere length and cellular senescence among other markers; Pop = patients of all age groups; study design = cohort, cross-sectional, or case-control study. Each section of the review was done and reported according to the Preferred Reporting Items for Systematic Reviews and Meta-Analyses (PRISMA) guideline using the published protocol (registration number = CRD42022298499) (Moher et al., 2009).

### Data Sources and Search Strategy

Google Scholar, PubMed, and Cochrane Library electronic databases were searched for relevant studies published from August 1970 to November 2021; last search was performed on 24 November 2021. Additional studies were searched manually from the lists of references of included studies. The following search terms were used for comprehensive search of available relevant studies from the electronic databases: (A) Google Scholar search terms: allintitle: “hypoxia” OR “sleep apnea” OR “intermittent hypoxia” OR “hypoxia-normoxia” OR “interval hypoxia” OR “hypoxic-normoxic” OR “hypoxia-hyperoxia” OR “hypoxic-hyperoxic” AND “aging” OR “ageing” OR “anti-aging” OR “anti-ageing”. (B) PubMed search terms: ((((((((hypoxia[MeSH Terms]) OR sleep apnea[MeSH Terms]) OR interval hypoxia[MeSH Terms]) OR intermittent hypoxia[MeSH Terms]) OR hypoxia-normoxia[MeSH Terms]) OR hypoxic-normoxic[MeSH Terms]) OR hypoxia-hyperoxia[MeSH Terms]) OR hypoxic-hyperoxic[MeSH Terms]) AND ((((aging[MeSH Terms]) OR aging[MeSH Terms]) OR anti-aging[MeSH Terms]) OR anti-ageing[MeSH Terms]). (C) Cochrane Library: Trials matching Hypoxia in Record Title OR Sleep apnea in Record Title AND Aging in Record Title – in Trials (Word variations have been searched).

### Study Selection and Eligibility Criteria

A two-step selection strategy was employed to select eligible articles after duplicate studies were excluded. In the first step, titles and abstracts of identified studies were screened for eligible original articles, which are published in English based on our inclusion and exclusion criteria. In the second step, full-text articles of the selected studies were downloaded and reviewed according to the following inclusion and exclusion criteria.

#### Inclusion Criteria

Studies were conducted to assess the effects of IHT (IHNT and IHHT), and for the cell culture model, continuous hypoxia training (CHT) on aging and age-related diseases was performed using the described protocol. Studies were conducted on sleep apnea (SA) such as obstructive sleep apnea (OSA), central sleep apnea (CSA), and mixed sleep apnea (MSA) as non-regulated, pathogenic hypoxia on aging and age-related diseases with the described protocol. Experimental studies were performed in human and cell line models. Studies conducted to assess the effect of IHT or SA on potential anti-aging biomarkers including telomere shortening, cellular senescence, genomic instability, epigenetic changes, oxidative stress, antioxidants, mitochondrial dysfunction, decreased autophagy, decreased proteostasis, stem cell exhaustion, altered intercellular communication, and deregulated nutrient-sensing were included in this systematic review.

#### Exclusion Criteria

Publications other than original article such as conference proceedings, review articles, case series, editorial, commentary, and opinion; animal studies; drug efficacy trials; articles with no full text access, and studies published in other languages than English were excluded from the review.

### Data Extraction

Data were extracted using a structured data sheet in Excel. The authors’ name, publication year, country of the study, study design, interval hypoxia protocol, criteria of sleep apnea, aging biomarkers, main findings, conclusions of the authors, safety issues, and methodological quality assessments or scores among other information associated with the review question were extracted. Authors (BT and BK) independently screened the identified studies and extracted the data. The data extracted by two authors from the selected studies were compared for consistency. The discrepancies were agreed and solved after rechecking the data and through discussion on the information. Studies were grouped as IHNT, IHHT, and OSA as follows: studies with the training program consisting of 15–24 sessions of intermittent hypoxia (10–12% O_2_)–normoxia (21% O_2_) were grouped as IHNT studies, while studies with the training program consisting of 15–24 sessions of intermittent hypoxia (10–12% O_2_)–hyperoxia (30–40% O_2_) were grouped as IHHT studies. Moreover, studies that investigated the effect of chronic sleep-related breathing disorder characterized by recurrent pauses in breathing on aging markers and age-related diseases were grouped as OSA studies. The systematic review process and main findings from the comprehensive literature search are summarized in figures and tables.

### Risk of Bias Assessment

Two independent reviewers (BT and BK) did the assessment of the risk of bias for the included studies. The assessment of the methodological quality for interval hypoxia training studies was performed according to the Cochrane tool for risk of bias assessment checklist. Each section was graded as high, unclear, or low: low grade indicates the highest quality (Higgins et al., 2011). Similarly, the methodological quality of sleep apnea studies was assessed based on the Newcastle Ottawa Scale (NOS) (Stang, 2010): the overall quality score was assessed out of nine points for cohort and case–control studies, while the highest overall quality score for cross-sectional studies was seven. Nine and seven cores indicate the highest quality for cohort/case-control and cross-sectional studies, respectively.

### Data Analysis

In this systematic review, we summarized the data of the primary studies on hypoxia intervention groups and sleep apnea cases by using a qualitative comparison with the control groups (normoxia and individuals without SA). However, pooling of the quantitative data from the individual studies in a meta-analysis was not possible due to the methodological variations among the primary studies.

## Results

### Search Results

**FIGURE 1:**
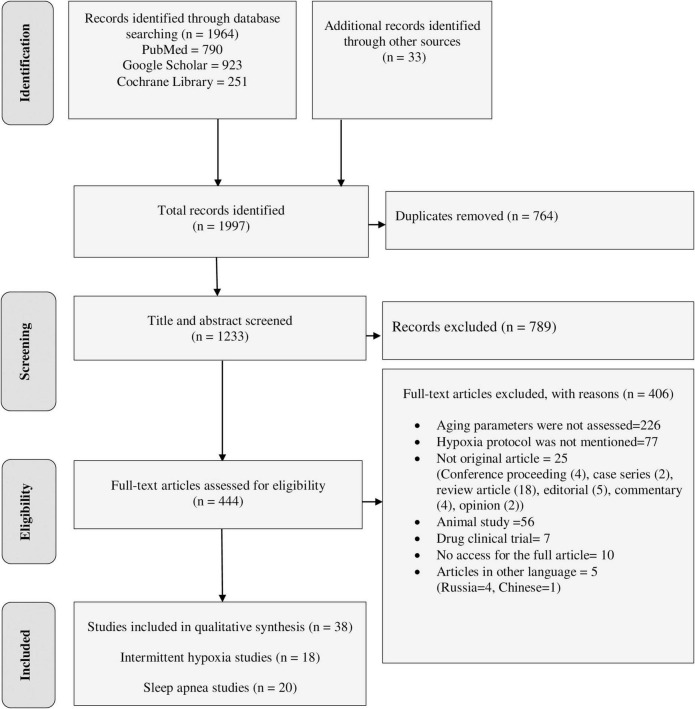
PRISMA flow diagram shows the searching strategy and screening of eligible studies at different levels of the review process (Moher et al., 2009).

The final databases search yielded 1,964 relevant studies (790 from PubMed, 923 from Google Scholar, and 251 from Cochrane Library). Manual searching from reference lists of eligible studies also identified 33 additional articles. The majority of the identified studies were excluded from this review for the reasons indicated in Figure 1. Finally, a total of 38 studies were eligible for data extraction and included in this systematic review.

### Characteristics of Included Studies

Of the total 38 studies, 18 studies investigated the effect of IHT on aging and age-related diseases, while 20 studies reported the effect of SA on aging and age-related diseases. Of the 18 IHT studies, 13 studies were done in humans and 5 studies were conducted in cell culture model. Of the 13 studies done in humans, eight studies were conducted on IHNT, one study was conducted on both IHNT and CHT (Table 1 and Supplementary Table 1), two studies were done on IHHT, and two studies were done on both IHHT and IHNT (Table 2 and Supplementary Table 2). Of the 5 studies done in the cell culture model, three studies were done on CHT and two studies were done on IHNT (Table 3 and Supplementary Table 3). All the twenty SA studies were conducted on the effect of OSA on aging and age-related diseases (Tables 4, 5 and Supplementary Tables 4, 5).

**TABLE 1:** The effects of intermittent hypoxia-normoxia training (IHNT) on aging markers and age-related diseases.

Author, year (ref.)	Condition	Age in years	Study design	Type of hypoxia	Hypoxia protocol	Aging markers	Results	Conclusion	Safety issues
Bao et al., 2019	Dizziness	35–62	RCT	IHNT	10% O_2_ for 50 min, 5 times per week for 4 weeks. Each session has 5 cycles of 10% O_2_ for 5 min/room air for 5 min.	Dizziness	There were significant differences between IHNT group and control group in DHI, ABC, VVAS scores and attack frequencies of dizziness in the end of 4th week.	IHNT could improve dizziness after intervention in the end of 4th week. IHNT could be the effective method for releasing dizziness.	Safe
Costalat et al., 2018	Overweight and obese	Mean, 56.2	RCT	IHNT	10% O_2_ repeated cycles with normoxia for 70 min, 10 sessions, 5 days/week for 2 weeks	Blood glucose and lactate	Glucose decreased and Lactate increased following a single IHNT session, but no sustained change after 10 sessions of IHNT occurred	IHNT appears to be a safe and effective non-pharmacological method of reducing key cardiovascular risk factors associated with metabolic disorders.	Safe
						HDL and LDL	LDL, LDL/HDL were all significantly decreased after 10 sessions.		
						SBP and DBP	SBP was significantly decreased after 10 sessions. No significant effects on DBP		
						RMSSD and DFAα1	No significant effects on heart rate, RMSSD, and DFAα1		
Faulhaber et al., 2015	COPD	mean, 50 (IHHT), 55 (control)	RCT	IHNT	12–15% Fi O_2_ for 3 weeks. 5 sessions/week, each consisting of 3–5 cycles, each cycle 3–5 min with 3-min breaks between cycles.	SBP and DBP, heart rate	During IHNT, no between-group differences were detected for blood pressure or rate pressure product values. Changes in heart rate were significantly different between groups in the course of the 3 weeks, with *post hoc* differences only in week 3.	IHNT resulted in specific and moderate heart rate and blood pressure responses, and did not provoke a progressive increase in blood pressure.	Safe
Muangritdech et al., 2020	Hypertensive patients	Mean range, 47–51	RCT	IHNT	Eight events of FI O_2_ 14%, and FI O_2_ 21% for 6 weeks. Each cycle consisted of 8 cycles × 3-min hypoxic air alternating with 3 min normoxic air.	Blood pressure	a significant decrease of the SBP in both IHNT at rest (IHR) and during excessive (IHT) groups at days 2 and 28 post-intervention, respectively.	IHNT may act as an alternative therapeutic strategy for hypertension patients probably through elevation of NOx and HIF-1α production.	Not mentioned
						Plasma NOx	IHR and IHT had increased NOx. At 2 days post-intervention, NOx was negatively correlated with SBP in IHT.		
						HIF-1α	IHR and IHT had increased HIF-1α. At 2 days post-intervention, HIF-1α was negatively correlated with SBP in IHT.		
						MDA levels	After 6 weeks of IHNT, MDA decreased to similar levels in the IHR and IHT compared to the control.		
						The time to complete a 6-min walk.	Improved walk distance was maintained at day 28 post-intervention, compared to the control, for both the IHR and IHT.		
Schega et al., 2013	Healthy	60–70	RCT	IHNT	Three sessions/week, 1 session for 1 h, hypoxic for 10 min and normoxia for 5 min. In the first 2 weeks, 90% SpO_2_. In the 3rd week, 85% SpO_2_. and 80% SpO_2_ for the next 3 weeks.	Cognitive performance	A time × group effect was observed by d2 test. No interaction effect was discovered by ZVT.	IHNT combined with physical exercise augments the positive effects of exercise on cognitive performance and QoL in elderly humans.	Not mentioned
						Quality of life (QoL)	An interaction effect was not found in the SF-12. But, an interaction effect was observed for sleep quality by PSQI.		
Schega et al., 2016	Healthy	60–65	RCT	IHNT	Hypoxia for 90 min followed by an aerobic training on bicycle ergometers for 30 min under ambient air. 3 times/week for 4 weeks. In the first week, the SpO2 between 90 and 85% and from the second to the fourth week 80%.	Physical performance	Increases the time to exhaustion	IHNT seems to be beneficial to enhance hematological parameters, physical performance and cognitive function in older people. The current hypoxic-dose was not able to enhance the serum BDNF-level or⋅VO_2_ max.	Not mentioned
						Cognitive function	An augmented and sustainable improvement in cognitive function		
						Hematological parameters (RBC, Hgb, Hct)	Increases in the values of hematological parameters		
						Serum BDNF-level.	In both groups, the⋅VO_2_ max and serum BDNF-level did not increase.		
Tobin et al., 2020	Healthy	Mean, 67	Uncontrolled CT	IHNT and CHT	Three phases of training for 3 weeks: 2 weeks normal air and normoxia, 5 days IHT hypoxia (SpO_2_ of 85%) for 70 min. After a 5-month washout period, CHT (SpO_2_ = 85%, for 70 min).	RBC, Hgb Hct	RBC, and Hgb only increased by day 5 of IH treatment compared to day 5 baseline values and day 5 sham values. Hct did not change in either Sham-IH or Sham-CH regardless of the baseline values.	These results revealed that inherent differences in the IH and CH hypoxic patterns could provide crucial components required to trigger hematological changes in senior individuals, without eliciting immunological stress responses or damaging the myocardium.	Safe
						Percentage of reticulocytes (% Retics),	% Retics did not change in either Sham-IH or Sham-CH regardless of the baseline values.		
						The OFF-score	OFF-score value increased only during the final day of IH treatment.		
						S-IgA, cortisol, cTnT	No difference was observed in S-IgA, cortisol or cTnT following IH or CH.		
Törpel et al., 2019	Healthy	18–35 (young) and 60–75 (old)	RCT	IHNT	The intensity of hypoxia was adjusted for 3 h with two approaches: (1) FiO_2_ 13.5% (H-ext), and (2) the FiO_2_ (H-int) was individually adjusted to an SaO_2_ of the blood of 80%.	EPO	EPO increased significantly after 180 min in both cohorts (young and old) and in both investigations (H-ext and H-int).	After 180 min hypoxia, EPO increases significantly in both age cohorts. The amount of EPO expression is significantly higher in young people during the same internal intensity of hypoxia than in old people.	Safe
Zhang et al., 2019	Lung cancer	Mean 62 (HPC), 63 (control)	RCT	IHNT	FiO_2_ was initially set at 60%, and in cases of saturation of pulse oxygenation (SpO_2_) less than 92%, FiO2 was increased to 100%. Three cycles of 5-min hypoxia and 3-min ventilation applied to the non-dependent lung served as the HPC intervention.	PaO_2_/FiO_2_ ratio, and pulmonary function.	HPC significantly increased the PaO_2_/FiO_2_ ratio compared with the control at 30 min after one-lung ventilation and 7 days after operation. Compared with the control, it also significantly improved postoperative pulmonary function.	HPC improves postoperative oxygenation, enhances the recovery of pulmonary function, and reduces the duration of hospital stay in patients undergoing thoracoscopic lobectomy.	
						Postoperative pulmonary complications.	No significant differences between groups were observed in the incidence of pulmonary complications or overall postoperative morbidity.		
						Duration of hospital stay.	Markedly reduced the postoperative hospital stay duration.		

*IHNT, intermittent hypoxia-normoxia training; IHT, intermittent hypoxia training; RCT, randomized controlled clinical trial; DHI, the Dizziness Handicap Inventory; ABC, Activities-specific Balance Confidence Scale; VVAS, Vertigo Visual Analog Scale; HDL, high-density lipoprotein; LDL, low-density lipoproteins; SBP, systolic blood pressure; DBP, diastolic blood pressure; RMSSD, root mean square of successive R–R interval differences; DFAα1, short-term fractal scaling exponent; COPD, chronic obstructive pulmonary diseases; SaO_2_, O_2_ saturation; NOx, nitric oxide metabolites; HIF-1α, hypoxia-inducible factor-1alpha; MDA, malondialdehyde: QoL, quality of life; SF-12, The Medical Outcomes Study Short-Form 36-Item Health Survey; PSQI, Pittsburgh Sleep Quality Index; BDNF, brain-derived eurotrophic factor; S-IgA, secretory immunoglobulin A; RBC, red blood cells; Hgb, hemoglobin; Hct, hematocrit; cTnT, cardiac troponin T; EPO, erythropoietin; CHT, continuous hypoxia training.*

**TABLE 2:** The effects of intermittent hypoxia-hyperoxia training (IHHT) on aging markers and age-related diseases.

Author, year (ref.)	Condition	Age in years	Study design	Type of hypoxia	Hypoxia protocol	Aging markers	Results	Conclusion	Safety issues
Dudnik et al., 2018	Cardiac patients with comorbidities	Mean 66 (IHHT), 65 (control)	RCT	IHHT	15 sessions hypoxia (11–12% O_2_)—hyperoxia (30–33% O_2_) for 5 weeks: 3 sessions/week, 5–7 hypoxic periods of 4–6 min, 3 min hyperoxic recovery.	CRF	CRF in the IHHT group was not significantly different compared with the control group. Systolic and diastolic blood pressures were not significantly different between groups after treatment.	IHHT might be a suitable option for older patients who cannot exercise. A 5-week IHHT is as effective as an 8-week exercise program in improving CRF, without hematological changes.	Safe
						Hgb, RBC, reticulocyte	Hgb content was not significantly different between groups. RBC and reticulocytes did not change pre/post interventions in both experimental groups.		
Glazachev et al., 2017	coronary artery disease (CAD)	43–83	Non-RCT	IHHT	15 sessions hypoxia (10% O_2_)- hyperoxia (30% O_2_). 3 sessions/week, 5–7 hypoxic periods lasting 4–6 min, with 3-min hyperoxic recovery.	Exercise performance	The IHHT showed improved exercise capacity, reduced systolic and diastolic blood pressures, enhanced left ventricle ejection fraction, but only at 1-month follow-up.	IHHT is associated with improved exercise tolerance, healthier risks factors profile, and a better quality of life. The study also suggests that IHHT is as effective as an 8-week standard rehabilitation program in CAD patients.	Safe
						Blood markers (RBC, Hgb, reticulocyte); metabolic profiles (total cholesterol, LDL and HDL, triglycerides, and glucose)	Hgb and glycemia were unchanged after IHHT, but glycemia was significantly lower at the 1-month follow-up. Total cholesterol and LDL were lower after IHHT. At the 1-month follow-up total cholesterol was similar to pretreatment. Reticulocytes were significantly higher in the IHHT at the end of treatment and at 1-month follow-up.		
						Quality of life	The SAQ profile was improved and not significantly different to the control after standard rehabilitation. The IHHT was compared to the control at 1-month follow-up, and no differences were found.		
Serebrovska et al., 2019	Prediabetic patients	51–74	RCT	IHHT and IHNT	15 sessions IHHT and IHNT, 5 times/week for 3 weeks. Each session consisted of 4 cycles of 5 min of 12% FiO_2_ followed by 3 min of 33% O_2_ in nitrogen or 5 min of normoxia.	Serum total cholesterol, HDL, LDL, and triglycerides	The study showed the same positive effect of IHNT and IHHT: decreased total blood cholesterol and LDL; and an equally smaller drop in SpO2 during acute hypoxic test. Improved parameters persisted 1 month after training termination in both groups.	One of the advantages of IHHT over IHT observed in this study could be some reduction in the duration of the sessions due to shortening re-oxygenation periods.	Not mentioned
						Plasma glucose concentrations	The study showed the same positive effect of IHHT and IHNT: equal reduction of serum glucose concentrations, both fasting and 2 h of OGTT. Improved parameters persisted 1 month after training termination in both groups.		
Susta et al., 2020	Healthy	18–24	Uncontrolled CT	IHHT and IHNT	FIO2 11% for up to 7 min followed by 3–5 min of exposure to normoxia (room air) or hyperoxia, FIO2 30–35%	Oxidative stress (concentration of hydroperoxides)	Oxidative stress was similar after IHN and IHH exposures compared with baseline values.	Hypoxia (IHN and IHH) cause neither pronounced oxidative stress nor antioxidant capacity impairment in healthy humans.	Safe
						Antioxidant capacity	The antioxidant capacity was also similar between experimental groups after both modalities of exposure.		

*IHHT, intermittent hypoxia-hyperoxia; IHNT, intermittent hypoxia-normoxia training; RCT, randomized controlled clinical trial; CRF, cardiorespiratory fitness; RBC, red blood cells; Hgb, hemoglobin; Hct, hematocrit; SAQ, Seattle Angina Questionnaire.*

**TABLE 3:** The effects of IHNT and CHT on aging markers and age-related diseases using cell culture.

Author, year (ref.)	Targeted conditions	Cell line	Study design	Type of hypoxia	Hypoxia protocol (for cases)	Aging markers	Results	Conclusion	Safety issues
Casciaro et al., 2020	Cellular aging	hAFSCs	Non-RCT	CHT	1% O_2_ for 5 weeks up to 8–9 passages.	Stemness properties (mRNA levels of Oct4 upregulation and protein expression of SSEA4)	1% O_2_ extends stemness	Low oxygen concentrations might improve the generation of functional hAFSCs for therapeutic use by delaying the onset of cellular aging.	Not reported
						Proliferative ability	1% O_2_ extends proliferative features		
						Induction of senescence-associated markers	1% O_2_ delays induction of senescence-associated markers.		
						Changes in metabolism and resistance to stress	Hypoxic hAFSCs activate a metabolic shift and increase resistance to pro-apoptotic stimuli.		
						Osteogenic differentiation	Cells at low oxygen remain capable of osteogenesis for prolonged periods of time		
Damiani et al., 2021	Skin aging	HDF	Non-RCT	CHT	5% O_2_. HDF were passaged at 80% confluence. Cells were serially cultured until enough cells were obtained for all experiments.	Cellular proliferation rate	Increased cell proliferation under 21% O_2_ compared to 5% O_2_	The 21% O_2_ impose a mild oxidative stress on HDF which accelerates the aging process in culture compared to 5% O_2_ where the underlying level of oxidative stress is reduced. cells grown under normoxia undergo a “stress-induced premature senescence” when compared to their matched counterparts grown under hypoxia. The modulation of miR-181a to different oxygen tensions and its potential role in altering the expression of antioxidant genes could represent an important molecular event in skin aging.	Not reported
						Intracellular ROS	Lower levels of intracellular ROS in cells at 21% O_2_ compared to those at 5% O_2_.		
						Mitochondrial superoxide anion generation	Higher levels of mitochondrial superoxide anion in cells at 21% O_2_ compared to at 5% O_2_		
						CoQ10 level and oxidative status	Total coenzyme Q10 levels decrease with cell passages and increase with oxygen tension.		
						Total glutathione (GSH + GSSG)	Total glutathione levels reduce under Low oxygen tension		
						Single and double-strand DNA damage	DNA damage increased under 21% O_2_ vs. 5% O_2_.		
						β-galactosidase activity, p16, CAT, SOD1, SOD3, MMP1, and COL1A1 genes expression	Higher levels of SOD1 and SOD3, upregulation of MMP1 and downregulation of COL1A1 under 21% O_2_ vs. 5% O_2_.		
Minamino et al., 2001	Vascular disorder	VSMC	Non-RCT	CHT	1% O_2_ until 10 passages.	Telomerase activity	Chronic hypoxia can prolong the growth of human VSMC by inducing telomerase activity and telomere stabilization. Hypoxia induced phosphorylation of the telomerase catalytic component (TERT) and sustained high levels of TERT protein expression in VSMC compared to normoxia.	Hypoxic induction of telomerase activity could be involved in long-term growth of VSMC and may thus contribute to human vascular disorders.	Not reported
Tantingco and Ryou, 2020	Ischemic stroke	Mice microglia, EOC20 cells	Non-RCT	IHNT	Three days IHT consisting of 5–8 daily, 5–10 min cycles of hypoxia (3.5–4% O_2_) with intervening 4-min re-oxygenation.	Cell viability	Intermittent hypoxic training protects the microglia from oxygen–glucose deprivation/re-oxygenation stress.	Due to the effect of intermittent hypoxic training on the microglia phenotype, intermittent hypoxic training could be considered as an effective intervention in the treatment or rehabilitation program for the ischemic stroke victims.	Not reported
						TLR2 proteins content	The TLR2 protein content was significantly elevated in the oxygen–glucose deprivation and re-oxygenation group, and intermittent hypoxic training lowered it to normoxia level.		
						Anti-inflammatory cytokines (IL-10 and IL-4)	IL-10 and IL-4 were significantly increased in the intermittent hypoxic training groups.		
						Reactive oxygen species (ROS)	Intermittent hypoxic training lowers the ROS generation		
						Phagocytic activity	Intermittent hypoxic training increases phagocytic activity (about 12-fold) vs. normoxia.		
						Cell phenotype	Intermittent hypoxic training regulates the polarization of the microglial phenotype toward anti-inflammatory type M2.		
Polonis et al., 2020	OSA	HWPs	Non-RCT	IHNT	9 cycles of IH (30 min of 21% O_2_ followed by 30 min of 0.1% O_2_) per day for up to 7 days	Senescence in HWPs	A higher prevalence of cells positive for senescence-associated β-galactosidase activity was also evident with chronic IH exposure.	This study identifies chronic IH as a trigger of senescence-like phenotype in preadipocytes.	Not reported

*hAFSCs, human amniotic fluid stem cells; CHT, continuous hypoxia training; IHT, intermittent hypoxia training; IH, intermittent hypoxia; non-RCT, non-randomized controlled trial; HDF, human dermal fibroblasts; VSMC, vascular smooth muscle cell; HPLC, high performance liquid chromatography; qPCR, quantitative polymerase chain reaction; ELISA, enzyme linked immunosorbent assay; OSA, obstructive sleep apnea; HWPs, human white preadipocytes; ROS, reactive oxygen species; TLR2, toll-like receptor 2.*

**TABLE 4:** The effects of sleep apnea on aging markers and age-related diseases.

Author, year (ref.)	Condition	Age in years	Study design	Total quality score^#^	Type of sleep apnea	Sleep apnea criteria	Aging markers	Results	Conclusion
Addison-Brown et al., 2014	SA	47–93	Case-control		OSA	High risk of OSA based on the Berlin Sleep Questionnaire.	Cognitive function	Those at high risk for OSA had significantly lower cognitive scores. However, some of the associations were age-dependent. Differences in cognition between those at high and low OSA risk were most pronounced during middle age, with attenuated effects after age 70 years.	Authors able to confirm OSA’s effect on cognition, depressive symptoms and HRQoL. They also found differential effects based on age, with more detrimental correlates of OSA in younger versus older adults, particularly in terms of mood and HRQoL.
				8			Depression	Those at high risk for OSA had significantly higher depressive symptoms.	
							HRQoL	Those at high risk for OSA had significantly lower HRQoL. However, some of the associations were age-dependent. Differences in quality of life between those at high and low OSA risk were most pronounced during middle age, with attenuated effects after age 70 years.	
Ayalon et al., 2010	SA	25–59	Case-control	6	OSA	AHI >10.	Cognitive performance and brain activation	Middle-aged patients with OSA showed reduced performance for immediate word recall and slower reaction time during sustained attention. For both tasks, decreased activation was detected for middle-aged sleep apnea relative to the other groups in task-related brain regions.	The presence of both sleep apnea and increasing age overwhelmed the brain’s capacity to respond to cognitive challenges with compensatory recruitment and to maintain performance.
Duffy et al., 2016	SA, and at risk for dementia	Mean, 65	Cross-sectional	7	OSA	SA was determined based on O_2_-desaturation and AHI, rapid eye movement sleep, and non-rapid eye movement sleep.	GSH and creatine	Increased levels of GSH/Cr were associated with lower oxygen desaturation and more severe apnea-hypopnea index scores during rapid eye movement sleep. In addition, ACC GSH/Cr correlated with poorer executive functioning (i.e., response inhibition and set shifting).	Markers of nocturnal hypoxemia and sleep disordered breathing (SDB) are associated with cerebral oxidative stress in older people at-risk for dementia, suggesting a potential mechanism by which SDB may contribute to brain degeneration, cognitive decline, and dementia.
Edwards et al., 2014	SA	20-40 (young) and >60 (old)	Case-control	7	OSA	OSA patients with AHI > 10 events/h and were treated with CPAP for more than 5 h per night for at least 2 months prior to enrollment.	Pharyngeal anatomy/collapsibility	In comparison with younger patients with OSA, older patients had a more collapsible airway.	The data suggest that airway anatomy/collapsibility plays a relatively greater pathogenic role in older adults, whereas a sensitive ventilator control system is a more prominent trait in younger adults with OSA.
							Loop gain (LG)	In comparison with younger patients with OSA, older patients had a lower LG	
							UAG	It was similar between groups.	
							RAT	It was similar between groups.	
Khan et al., 2011	SA	>65	Cohort	9	OSA	OSA was diagnosed when AHI was >5 events/h.	Hct and Hgb	Hct changed significantly post OSA treatment. The change in Hgb after OSA treatment was not significant. However, the change in Hgb was large enough to reach WHO standards for AoA. Hct changed significantly among both men and women.	There was no AOA before OSA treatment. But there was AOA 1 year after OSA treatment. Authors believe OSA inflammatory processes interact with OSA hypoxia-induced erythropoiesis.
Kritikou et al., 2014	SA	Mean 42–66	Case-control		OSA	AHI > 10 events/h of sleep for females and >15 events/h for males.	Sleepiness	Apnoeic males were significantly sleepier than controls. CPAP improved subjective sleepiness.	OSA is associated with sleepiness, inflammation and insulin resistance, even in non-obese males, and this association is stronger in males than in females. Short-term CPAP does not improve the inflammatory/metabolic aberrations in OSA.
				8			IL-6	Apnoeic males had significantly higher IL-6 than controls. Apnoeic females had IL-6 similar to controls. CPAP did not change IL-6	
							TNFR-1	No significant difference was observed in TNFR-1 values. CPAP did not change TNFR-1	
							Leptin and adiponectin	Apnoeic males had significantly higher leptin than controls. Apnoeic females had leptin and adiponectin similar to controls. CPAP did not change leptin and adiponectin	
							hsCRP	Apnoeic males and females had significantly higher hsCRP. CPAP did not change hsCRP.	
							Fasting glucose and insulin levels	Apnoeic males had significantly higher insulin resistance than controls. Apnoeic females had insulin resistance similar to controls. CPAP did not change insulin resistance.	
Sajkov et al., 1998	SA and tetraplegia	18–60	Case-control	7	OSA	AHI > 15 per hour of sleep.	Neuropsychological function (e.g., memory, perception, attention, and concentration)	The neuropsychological functions most affected by nocturnal desaturation were verbal attention and concentration, immediate and short-term memory, cognitive flexibility, internal scanning and working memory.	SA impairs daytime cognitive function in tetraplegia patients, particularly attention, concentration, memory and learning skills. Cognitive disturbances resulting from SA might adversely affect rehabilitation in patients with tetraplegia.
Vgontzas et al., 2019	SA	20–80	Cohort	9	OSA	Mild OSA = AHI 5–14.9 events/h, and moderate OSA = AHI 15–29.9 events/h. All cases were without hypertension at the baseline.	Incident hypertension	Mild-to-moderate OSA was significantly associated with increased risk of incident hypertension. Importantly, this association was modified by age; while strong in young and middle-aged adults, the association was lost in adults older than 60 years. Furthermore, the association of mild-to-moderate OSA with components of metabolic syndrome was strongest in young and middle-aged adults.	Mild-to-moderate OSA, even when asymptomatic, is associated with increased risk of incident hypertension, but the strength of association significantly decreases with age.
Weihs et al., 2021	SA	Mean 52.5	Cross-sectional	7	OSA	Mild to severe OSA (AHI and ODI ≥ 5) and severe OSA (AHI and ODI ≥ 15).	A score quantifying age-related brain patterns in 169 brain regions	AHI and ODI were both positively associated with brain age. The effects remained stable in the presence of various confounders such as diabetes and were partially mediated by the white blood cell count, indicating a subclinical inflammation process.	The study reveals an association between OSA and brain age, indicating subtle but widespread age-related changes in regional brain structures.
Yim-Yeh et al., 2010	SA	18–70	Cross-sectional	6	OSA	AHI>10/h.	Vascular function, and arterial stiffness	FMD was impaired in patients with OSA. OSA did not significantly influence vascular function in the skin microcirculation. The augmentation index, a measure of arterial stiffness, was similar between the OSA and control groups, OSA independently predicted the augmentation index in men only.	In obesity, both OSA and aging impair endothelial function and increase arterial stiffness.

*^#^Total quality score out of 9 for cohort/case-control and out of 7 for cross-sectional; SA, sleep apnea; OSA, obstructive sleep apnea; HRQoL, health-related quality of life; AHI, apnea–hypopnea index; fMRI, functional magnetic resonance imaging; CPAP, continuous positive airway pressure; ESS, Epworth Sleepiness Scale; MSLT, multiple sleep latency test; PVT, the psychomotor vigilance test; IL-6, interleukin-6; TNFR-1, tumor necrosis factor receptor-1; hsCRP, high-sensitivity C-reactive protein; ODI, oxygen desaturation index; FMD, flow-mediated dilation; 1H-MRS, proton magnetic resonance spectroscopy; GSH, glutathione; UAG, upper airway muscle responsiveness/gain; RAT, respiratory arousal threshold.*

**TABLE 5:** The effects of sleep apnea on aging markers (leukocyte telomere length) and age-related diseases.

Author, year (ref.)	Conditions	Age in years	Study design	Total quality score^#^	Type of sleep apnea	Sleep apnea criteria	Aging markers	Results	Conclusion
Barceló et al., 2010	SA	49.5	Case-control	7	OSAS	AHI > 10	LTL	TL was significantly shorter in patients with OSAS than in controls. This difference persisted after adjustment for age, presence of cardiovascular and metabolic changes. TL was not related to the severity of OSAS.	TL in circulating leukocytes is shorter in patients with OSAS than controls. The mechanism of this observation is unresolved since it appears independent of chronological age, the severity of OSAS and/or the presence of cardiovascular or metabolic changes.
Carroll et al., 2019	SA	44–84	Cohort	9	OSA	AHI > 15	LTL	Severe obstructive sleep apnea was associated with shorter LTL. An exploratory analysis found that higher arousal index at Exam 5 was associated with greater LTL decline over the prior 10 years.	OSA was associated with shorter leukocyte telomere length. Individuals with high arousal frequency had greater leukocyte telomere attrition over the prior decade. These findings suggest that sleep apnea and sleep fragmentation are associated with accelerated biological aging.
Boyer et al., 2016	SA	46.8	Case-control	6	OSAS	AHI > 5/h	LTL	AHI and oxygen desaturation index were significantly related to telomere shortening.	Intermittent hypoxemia due to OSAS is a major contributor to telomere shortening in middle-aged men. Oxidative stress may explain this finding.
Kim et al., 2010	SA	5–10	Case-control	8	OSA	AHI > 1/hrTST	LTL	LTL was independently associated with AHI.	In pediatric OSA, LTL is longer rather than shorter. Children with OSA have reduced plasma catestatin levels and increased BP along with increased MRP 8/14 levels that exhibit AHI dependencies. Thus, catestatin and MRP 8/14 levels may serve as biomarkers for cardiovascular risk in the context of pediatric OSA. However, the implications of increased LTL in children with OSA remain to be defined.
							Catestatin	Children with OSA exhibited lower plasma catestatin.	
							MRP 8/14	Children with OSA exhibited higher MRP 8/14 levels than controls	
Kim et al., 2016	SA	45.6	Case-control	7	OSA	RDI ≥ 5	LTL	Significantly shortened TL was observed in the circulating leukocytes of the peripheral blood of OSA patients, and TL shortening was aggravated more acutely in an age- and BMI-dependent manner.	The results provided evidence that telomere length shortening or excessive cellular aging might be distinctive in circulating leukocyte of OSA patients.
Kwon et al., 2015	SA	58.9 years	Cohort	9	OSA	AHI ≥ 5	LTL	Sleep stability significantly reduced with shortened LTL in OSA patients.	The present study suggested that shorter LTL might contribute to reduced sleep stability by interacting with OSA severity due to the stress of chronic sleep fragmentation or invariant sympathetic activity by respiratory chemoreflex activation.
Polonis et al., 2019	SA	35.6 (cases) and 47.3 (controls)	Case-control	8	OSA	AHI ≥ 5	LTL	The OSA group had a higher likelihood of cancer but no clear evidence of an elevated incidence of MACE compared to the non-OSA group. There was no association between TL and MACE or cancer-risk.	The study warrants further investigation of any modulating effect of OSA on TL and the risk of MACE and cancer.
Polonis et al., 2017	SA	27–57	Case-control	7	OSA	AHI ≥ 5	LTL	There was no difference in telomere length between OSA and control group. The mean TL in moderate-to-severe OSA was significantly longer than in control group after adjustment for age, sex, BMI, hypertension, dyslipidemia and depression.	Moderate-to-severe OSA is associated with telomere lengthening. These findings support the idea that changes in TL are not unidirectional processes such that telomere shortening occurs with age and disease, but may be prolonged in moderate-to-severe OSA.
Riestra et al., 2017	SA	30–55	Case-control	9	OSA	High risk of having OSA if scores were positive for two or more of the three categories by Berlin questionnaire.	LTL	The study showed that LTL varied by OSA risk in women. Multiple linear regression analysis confirmed that women at higher risk for OSA presented shorter LTL compared to those at lower risk. These differences were not observed in men.	These findings suggest that OSA risk may contribute to the acceleration of cellular aging processes through telomere shortening.
Tempaku et al., 2016	SA	20–80	Case-control	8	OSAS	AHI > 5	LTL	LTL was significantly shorter in OSAS patients compared to controls.	The study indicates the presence of an association between LTL and OSAS and a significant impact of severity of OSAS in telomeres shortening.

*^#^Total quality score out of 9 for cohort/case-control and out of 7 for cross-sectional; SA, sleep apnea; OSA, obstructive sleep apnea; TL, telomere length; LTL, leukocyte telomere length; AHI, apnea–hypopnea index; RDI, respiratory disturbance index; qPCR, quantitative polymerase chain reaction; hrTST, hour of total sleep time; MRP, myeloid-related protein; ROS, reactive oxygen species; MACE, major adverse cardiac events.*

Among 18 studies on IHT, the study designs were randomized controlled clinical trial (RCT) for 10 studies, non-randomized clinical trial for 7 studies, and uncontrolled clinical trial for one study. Of the total 20 studies on OSA, 13 studies were case-control, 4 cohort, and 3 cross-sectional studies. The age groups of human study participants in the IHT studies range from 18 to 83 years (Tables 1, 2), while for SA studies the age groups of the study participants range from 5 to 93 years (Tables 4, 5).

### Quality Assessment

The results of the risk of bias assessment for 18 IHT studies showed that the risk of selective reporting bias, incomplete outcome data bias (long term), and incomplete outcome data bias (short term) were low in all studies. However, one study had unclear risk of allocation concealment bias, two studies had unclear blinding of outcome assessment bias (all case mortality), two studies in human and five studies in cell culture model had unclear risks of random sequence generation bias, one study had unclear and another one study had high risk of blinding of outcome assessment bias (patient reported). Moreover, two studies had high risks of blinding of participants and personnel bias. Similarly, the results of risk of bias assessment by NOS for the 20 OSA studies showed that the overall quality scores for cohort and case-control studies ranged from 6 to 9, while the overall quality scores ranged from 6 to 7 for cross-sectional studies. Overall, 7/20 OSA studies scored the highest quality scores (Tables 4, 5).

### The Effects of Intermittent Hypoxia-Normoxia Training and Intermittent Hypoxia-Hyperoxia Training on Age-Related Diseases

Randomized clinical trial studies have demonstrated the beneficial effects of IHNT and IHHT on age-related diseases including cardiovascular diseases, cognitive disorders, lung diseases, diabetes, hematological disorders, oxidative stress, inflammation, and quality of life (Tables 1, 2 and Supplementary Tables 1, 2).

#### Cardiovascular Diseases

A study aimed to investigate the effects of moderate IHNT on key cardio-metabolic risk factors in overweight and obese subjects showed decreased glucose and increased lactate levels following a single IHNT session, but no sustained change after 10 sessions of IHNT occurred. Conversely, LDL, LDL/HDL ratio, and SBP were all significantly decreased after 10 sessions (Costalat et al., 2018). During IHNT intervention (15 sessions), variations for heart rate and rate pressure product were significantly higher in the hypoxia group and tended to be increased for systolic blood pressure (Faulhaber et al., 2015). A study by Muangritdech et al. (2020) showed a significant decrease of the SBP in both IHNT at rest and IHNT during exercise groups at days 2 and 28 post-intervention, respectively, compared with control (Table 1). Dudnik et al. (2018) demonstrated the positive effect of IHHT on cardiorespiratory fitness compared with a standard exercise-based rehabilitation program, whereas no difference in blood pressure and in hemoglobin content was observed. In the study of Glazachev et al. (2017), IHHT also improved exercise capacity, enhanced left ventricle ejection fraction, reduced SBP and DBP, and reduced glycemia at 1-month follow-up. Angina was also significantly reduced after treatment and at 1-month follow-up (Table 2).

#### Cognitive Function and Physical Improvement

Bao et al. (2019) reported that dizziness scores and attack frequencies of dizziness were significantly decreased after IHNT intervention at the end of the 4th week. Moreover, Schega et al. (2013) suggested that an additional intermittent hypoxic training combined with physical exercise augments the positive effects of exercise on cognitive performance in elderly humans. IHNT also increases the values of the time to exhaustion in the load test and an augmented and sustainable improvement in cognitive function in older people (Schega et al., 2016) (Table 1).

#### Lung Diseases (Pulmonary Function)

A prospective randomized controlled trial study showed that hypoxic preconditioning (HPC) of patients with lung cancer undergoing elective thoracoscopic lobectomy significantly increased the PaO2/FiO2 ratio compared with patients without HPC at 30 min after one-lung ventilation and 7 days after operation. HPC also markedly decreased the duration of postoperative hospital stay and significantly improved postoperative pulmonary function (Zhang et al., 2019) (Table 1).

#### Hematological Parameters

Several studies reported the positive effects of IHNT and IHHT on hematological parameters. RBC, Hgb, and Hct increased after IHNT intervention (Schega et al., 2016). Similarly, RBC and Hgb increased by day 5 of IHNT treatment compared with day 5 baseline values and day 5 sham values. Likewise, Hct increased during the final day of IHNT treatment (Tobin et al., 2020). EPO increased significantly after 180 min of IHNT intervention in both old and young cohorts. EPO expression was significantly higher in young than in old people after 180 min of hypoxic exposure and 30 min afterward (Törpel et al., 2019) (Table 1).

#### Oxidative Stress and Inflammation

A study by Susta et al. (2020) measured oxidative stress and antioxidant capacity in healthy humans after being acutely exposed to both IHNT and IHHT. After IHNT and IHHT, neither oxidative stress nor antioxidant capacity was significantly different compared with baseline. Oxidative stress and antioxidant capacity were not significantly different between IHNT and IHHT groups. As a result, authors concluded that IHNT and IHHT cause neither antioxidant capacity impairment nor pronounced oxidative stress in healthy humans (Susta et al., 2020). An *in vitro* cell culture study by Tantingco and Ryou (2020) also reported that intermittent hypoxic training protects the microglia from oxygen–glucose deprivation/re-oxygenation stress. ROS in the oxygen–glucose deprivation/re-oxygenation group is increased, but intermittent hypoxic training lowers the ROS generation by oxygen–glucose deprivation/re-oxygenation. Moreover, intermittent hypoxic training regulates the polarization of the microglial phenotype toward anti-inflammatory type M2 (Tantingco and Ryou, 2020).

#### Quality of Life and Diabetes

The findings of the study by Schega et al. (2013) suggested that an additional intermittent hypoxic training combined with physical exercise augments the positive effects of exercise on quality of life (QoL) in elderly humans (Schega et al., 2013). Similarly, Glazachev et al. (2017) showed that quality of life, risk factors profile, and exercise tolerance were improved in the IHHT group in the same manner as identified in the standard rehabilitation group immediately after the course and at 1-month follow-up. Authors also suggested IHHT as an effective intervention as a standard rehabilitation program in coronary artery patients. A placebo-controlled clinical trial study of the effects of IHNT and IHHT interventions on metabolic variables in prediabetes patients showed the positive effects of both types of training, namely, reduction of serum glucose level, at fasting point and 2 h after glucose tolerance test, decrease of total blood cholesterol and LDL, and increase in SpO_2_ during acute hypoxic test. Improved parameters persisted 1 month after training termination in both groups (Serebrovska et al., 2019) (Table 2).

### The Effects of Intermittent Hypoxia-Normoxia Training and Intermittent Hypoxia-Hyperoxia Training on Aging Markers

A cell culture study by Minamino et al. (2001) reported that the growth of human vascular smooth muscle cell (VSMC) could be prolonged by chronic hypoxia (1% O_2_) *via* inducing telomerase activity and telomere stabilization. Authors demonstrated hypoxia-induced sustained high levels of TERT protein expression and phosphorylation of the telomerase catalytic component compared with normoxia (Minamino et al., 2001). Another cell culture study by Casciaro et al. (2020) showed that hypoxia (1% O_2_) delays induction of senescence-associated markers (p16 and p21) expression and senescence-associated β-galactosidase, upregulates pluripotent marker (Oct4), activates a metabolic shift, and raises resistance to pro-apoptotic stimuli in human amniotic fluid stem cells. Similarly, Tantingco and Ryou (2020) showed that intermittent hypoxic training (3.5–4% O_2_) protects the microglia from oxygen–glucose deprivation/re-oxygenation stress. Hypoxia also preserves osteogenesis for extended periods of time, indicating a more youthful phenotype (Casciaro et al., 2020).

Damiani et al. (2021) demonstrated that aging markers such as β-galactosidase activity, p16 expression, and proliferation rate, as well as expression of miRNA-181a, were significantly increased in human dermal fibroblast under 21% O_2_ compared with those under 5% O_2_. Despite the positive effects on cell aging markers, incubation under 5% O_2_ was associated with higher levels of total GSH, mitochondrial superoxide anion, CoQ10, SOD1, and SOD3, lower levels of intracellular ROS, MMP1 expression, and DNA damage (Damiani et al., 2021). Muangritdech et al. (2020) showed that NOx and HIF-1α were upregulated after IHNT at rest and during exercise compared with the control group. The dynamics of NOx and HIF-1α were negatively associated with systolic blood pressure in IHNT during exercise 2 days post-intervention (Muangritdech et al., 2020). However, a cell culture study on human white preadipocytes (HWPs) demonstrated that chronic intermittent hypoxia at a very low oxygen concentration (0.1% O_2_) is related to a higher level of ROS and increased number of cells with nuclear localization of γH2AX and p16. A higher number of cells positive for senescence-associated β-galactosidase activity were also observed in chronic intermittent hypoxia exposure (Polonis et al., 2020).

In general, intermittent hypoxia of moderate level and duration provides a positive effect on several age-related parameters including quality of life, cognitive and physical functions, plasma level of glucose and cholesterol/LDL, systolic blood pressure, red blood cells, and inflammation. Moreover, moderate intermittent hypoxia induces TERT activity and telomere stabilization, delays induction of senescence-associated markers (p16 and p21) expression and senescence-associated β-galactosidase, upregulates pluripotent marker (Oct4), activates a metabolic shift, and raises resistance to pro-apoptotic stimuli (Table 3).

### The Effects of Obstructive Sleep Apnea on Age-Related Diseases

Several investigators have documented the effects of OSA on age-related diseases such as cardiovascular diseases, cognitive disorders, hematological disorder, oxidative stress, and inflammation (Table 4 and Supplementary Table 4).

#### Cardiovascular Diseases

A study by Vgontzas et al. (2019) reported that mild-to-moderate OSA was significantly related to a higher risk of incident hypertension and metabolic syndrome in young and middle-aged persons, but not in adults older than 60 years. Similarly, Kim et al. (2010) reported that vascular function impairment and arterial stiffness were associated with OSA in younger (≤50 years), but not in older, men. In children, OSA increased BP and decreased plasma catestatin levels together with increased multidrug-resistance-associated protein 8/14 levels (Kim et al., 2010).

#### Cognitive Function, Physical Improvement, and Quality of Life

Addison-Brown et al. (2014) assessed cognitive function in OSA high-risk patients with standardized fluency and recall measures. Patients at high risk for OSA had significantly reduced quality of life (depressive symptoms and health-related quality of life) and decreased cognitive scores. However, some of the associations were age-dependent. During middle age, differences in quality of life and cognition between high and low OSA risk were most pronounced, with attenuated effects after 70 years of age (Addison-Brown et al., 2014). Middle-aged OSA patients showed slower reaction time during sustained attention and decreased performance for immediate word recall compared with the middle-aged control, young control, and young sleep apnea. For both functions, reduced activation was detected for middle-aged OSA cases relative to the other groups in task-related brain regions (Ayalon et al., 2010). GSH/Cr level increased during lower oxygen desaturation and more severe apnea-hypopnea index (AHI) scores. Poorer executive functioning was also correlated with GSH/Cr level (Duffy et al., 2016). Moreover, men with OSA were significantly sleepier than controls, while women with OSA showed comparable objective sleepiness with controls (Kritikou et al., 2014).

According to Sajkov et al. (1998)’s report, neuropsychological variables in patients with tetraplegia were significantly correlated with measures of sleep hypoxia, but not with the apnea-hypopnea index and the frequency of sleep arousals. The most affected neuropsychological functions by sleep hypoxia were immediate and short-term memory, cognitive flexibility, verbal attention and concentration, internal scanning, and working memory. The authors concluded that sleep apnea is common in patients with tetraplegia and may be associated with significant oxygen desaturation. The oxygen desaturation weakens daytime cognitive function in these patients, mainly memory, learning skills, attention, and concentration. Cognitive disturbances resulting from OSA might adversely affect rehabilitation in patients with tetraplegia (Sajkov et al., 1998). A cohort study by Khan et al. (2011) showed that OSA patients did not suffer from anemia of aging when living without OSA treatment, whereas symptoms started to appear 1 year after treatment. Researchers suggest that OSA inflammatory progressions are related to OSA hypoxia-induced erythropoiesis (Khan et al., 2011).

#### Hematological Parameters

A study by Khan et al. (2011) investigated OSA and anemia of aging (AoA) coexistence, and OSA treatment-AoA interaction. The study showed that Hct changed significantly post OSA treatment. Moreover, Hct changed significantly among both men and women. The change in Hgb after OSA treatment was not significant. However, the change in Hgb was large enough to reach WHO standards for AoA (Table 4).

#### Oxidative Stress, Inflammation, and Diabetics

Increased levels of GSH/Cr were associated with lower oxygen desaturation and more severe apnea-hypopnea index scores during rapid eye movement sleep. In addition, GSH/Cr correlated with poorer executive functioning (Duffy et al., 2016). Serum concentration of hydrogen peroxide was also considerably higher in OSA patients, and it was closely related to the severity of OSA (Kim et al., 2016). Men with OSA had significantly higher IL-6, hsCRP, insulin resistance, and leptin than controls, while women with OSA had significantly elevated level of hsCRP; however, TNFR-1, IL-6, insulin resistance (Homeostatic Model Assessment index), adiponectin, and leptin were similar to controls (Kritikou et al., 2014) (Table 4).

### The Effects of Obstructive Sleep Apnea on Aging Markers

A number of studies have reported the effects of OSA on aging using mainly leukocyte telomere length (LTL) as a marker of aging (Table 5 and Supplementary Table 5). Barceló et al. (2010) reported that TL was significantly shorter in patients with OSA than in controls. This difference continued after adjustment for body mass index, age, glucose, cholesterol, uric acid and triglycerides levels, the presence of arterial hypertension, and smoking status. The severity of OSA was not related to LTL as assessed by nocturnal oxygen saturation, AHI, and daytime sleepiness (Barceló et al., 2010). However, severe OSA (OSA; AHI >30) was associated with shorter TL. A higher arousal index at exam 5 was correlated with higher LTL shortening over the prior 10 years (Carroll et al., 2019). TL was also independently associated with oxygen desaturation index (Boyer et al., 2016).

In OSA patients, TL of circulating leukocytes of the peripheral blood was significantly shortened, and TL shortening was aggravated more acutely in age- and BMI-dependent manner. The concentration of hydrogen peroxide and the TL of OSA patients were inversely correlated, and the severity of OSA was associated with excessive TL shortening. The findings indicate that TL shortening or extreme cellular aging might be typical in peripheral leukocytes of OSA patients. TL shortening may be a biomarker to reveal the problem of oxidative stress in the circulating blood of OSA patients (Kim et al., 2016). Increased narrow-band low-frequency coupling by relating with OSA severity showed a significant association with TL shortening. Particularly, in OSA patients, sleep stability significantly decreased with reduced TL (AHI ≥ 15) (Kwon et al., 2015). Polonis et al. (2019) reported that OSA patients had a higher likelihood of cancer but no clear evidence of an elevated incidence of major adverse cardiac events and no association with TL or cancer risk.

Telomere shortening was observed in women at higher risk for OSA compared with those at lower risk, independent of income, age, obesity, smoking, hypertension, alcohol consumption, and education. This change was not seen in men (Riestra et al., 2017). Tempaku et al. (2016) demonstrated that in OSAS patients, TL was significantly reduced compared with controls. There were negative associations between TL and desaturation index, AHI, wake after sleep onset, and respiratory disturbance index. LTL was directly related to basal minimum and maximum oxygen saturation, total sleep time, and sleep efficiency (Tempaku et al., 2016).

A J-shaped association was shown between TL and OSA severity. In moderate-to-severe OSA, the mean TL was significantly larger than that in the control group. These results strengthen the idea that variations in TL are not unidirectional processes such that TL reduction occurs with disease and age, but may be elongated in moderate-to-severe OSA (Polonis et al., 2017). In children with OSA, TL is increased rather than decreased (Kim et al., 2010) (Table 5).

A study that investigated the correlation between advanced brain aging and OSA revealed that oxygen desaturation index (ODI) and AHI were positively correlated with brain age. The correlations persisted stable in the presence of a number of confounders including diabetes, and the correlations were partially mediated by the white blood cell count, showing a subclinical inflammation process. These findings show a correlation between OSA and brain age, showing subtle but extensive age-related changes in regional brain structures (Weihs et al., 2021). Edwards et al. (2014) assessed the physiological traits responsible for OSA in a group of young and old patients with OSA to understand the pathological mechanisms underlying OSA. In comparison with younger patients with OSA, older patients with OSA had a more collapsible airway but lower Veupnea and a lower loop gain. These findings suggest that a sensitive ventilatory control system is a greater prominent trait in younger adults, while airway anatomy/collapsibility has a relatively higher pathogenic role in older adults with OSA (Edwards et al., 2014).

In general, intermittent hypoxia in OSA causes hypertension, metabolic syndrome, vascular function impairment, quality of life and cognitive scores reduction, advanced brain aging, and increase in insulin resistance, plasma hydrogen peroxide, GSH, IL-6, hsCRP, leptin, and LTL shortening.

### Safety of Intermittent Hypoxia-Normoxia Training and Intermittent Hypoxia-Hyperoxia Training Protocols

Several researchers have assessed the safety of IHNT and IHHT protocols when used as an intervention for age-related diseases and during investigations of their effects on aging markers (Tables 1, 2 and Supplementary Tables 1, 2). Studies included in this review and that assessed the safety of IHNT and IHHT protocols in human have shown that both IHNT and IHHT protocols are safe interventions to use in humans (Faulhaber et al., 2015; Glazachev et al., 2017; Costalat et al., 2018; Dudnik et al., 2018; Bao et al., 2019; Törpel et al., 2019; Susta et al., 2020; Tobin et al., 2020).

## Discussion

### Main Findings

In this review, we have systematically summarized the available literature on the effects of IHT (IHNT and IHHT) and OSA on aging and age-related diseases according to the standard PRISMA guidelines. The IHNT and IHHT have similar effects on aging markers and age-related diseases, while OSA creates different and even opposite results compared with IHNT and IHHT. The main influencing factors in all studied groups of hypoxia are intensity and duration of hypoxic exposure. IHHT and IHNT provide 3–5 training per week with 3–5 hypoxic episodes (2–5 min of duration, SaO2 > 86–88%). OSA is diagnosed when a person regularly suffers 5–10 episodes (approximately 2 min of duration, SaO2 < 86%). Unlike IHHT and IHNT, OSA occurs at night time in a supine position and in a state of sleep accompanied by hypercapnia. Similarly, a review by Navarrete-Opazo and Mitchell (2014) showed that the adverse and beneficial effects of intermittent hypoxia depend on its dose. The severity of hypoxemia, the duration of hypoxia, the number of cycles/day, the pattern of intermittent hypoxia (consecutive days vs. alternating days), and the total protocol duration are crucial factors that determine its effects and even direction. In intermittent hypoxia with high cycle numbers and/or severe hypoxic episodes, adverse effects are more common, while in intermittent hypoxia with low cycle numbers per day and/or mild to moderate hypoxic episodes, apparently beneficial effects are more common. As the result, the finding of the review suggests that low-dose intermittent hypoxia has considerable therapeutic potential to treat multiple clinical conditions (Navarrete-Opazo and Mitchell, 2014).

The most pronounced effects of IHHT and IHNT on age-related diseases are cognitive and physical improvement (Schega et al., 2013, 2016; Glazachev et al., 2017; Bao et al., 2019), increased quality of life (Schega et al., 2013; Glazachev et al., 2017), and downregulation of glucose and cholesterol (LDL) (Glazachev et al., 2017; Costalat et al., 2018; Serebrovska et al., 2019), especially in persons with metabolic disorders. Blood pressure, especially systolic blood pressure, decrease is another positive effect of IHHT and IHNT described in several studies (Costalat et al., 2018; Muangritdech et al., 2020). While a slight increase in EPO was mentioned in only one study, no effect on circulating red blood cells (Hb and Hct) was found in several articles (Törpel et al., 2019).

Intermittent and chronic hypoxia influence aging biomarkers *in vitro*. Chronic exposure to 1% O_2_ prolongs the proliferation of human VSMC, upregulates telomerase (Minamino et al., 2001), downregulates the expression of senescence-associated markers, activates a metabolic shift, and increases resistance to pro-apoptotic stimuli (Casciaro et al., 2020). In the same manner, incubation under 5% O_2_ caused significant downregulation of senescent markers expression, mitochondrial superoxide anion, total GSH, CoQ10, SOD1, SOD3, and reduced levels of intracellular ROS in comparison with 21% O_2_. Moreover, miRNA-181a expression, which is known to be upregulated in cellular cycle arrest, was significantly increased in cells at 21% O_2_ compared with those at 5% O_2_. Increased DNA damage, upregulation of MMP1, and downregulation of COL1A1 were also observed under 21% O_2_ compared with cells at 5% O_2_ (Damiani et al., 2021). Similarly, intermittent hypoxic training (3.5–4% O_2_) protects the microglia from oxygen–glucose deprivation/re-oxygenation stress (Tantingco and Ryou, 2020). However, a cell culture study on HWPs demonstrated that chronic intermittent hypoxia exposure at a very low oxygen concentration (0.1% O_2_) is associated with an increased generation of mitochondrial ROS along with increased prevalence of cells with nuclear localization of γH2AX and p16 and a higher prevalence of cells positive for senescence-associated β-galactosidase activity (Polonis et al., 2020).

When speaking of OSA, it changes almost all parameters listed above for age-related diseases and aging markers in a negative direction. OSA is responsible for the cognitive and physical decline, even for brain damage, as well as for increased blood pressure, cholesterol/LDL, glucose, and insulin resistance, and lower health-related quality of life (Sajkov et al., 1998; Ayalon et al., 2010; Addison-Brown et al., 2014; Kritikou et al., 2014). Moreover, OSA significantly shortened LTL, common marker of aging (Barceló et al., 2010; Boyer et al., 2016; Kim et al., 2016; Tempaku et al., 2016; Riestra et al., 2017; Carroll et al., 2019), and brain aging (Weihs et al., 2021). The pleasant exceptions from negative OSA effects are the increase of Hb/Hct and prevention of age-related anemia (Khan et al., 2011), and in pediatric (age group 5–10 years) OSA cases, LTL is longer rather than shorter (Kim et al., 2010).

The explanation of dose-dependent effects of intermittent hypoxia should be found more likely in mechanisms of action. It is well known that hypoxia causes cell damage followed by inflammation and HIF-1α accumulation (Chen and Gaber, 2021; Shao et al., 2021). Inflammation is described many times as a significant pathogenic factor of OSA (Khalyfa et al., 2018; Wang et al., 2022). At the same time, intermittent hypoxia in IHHT, IHNT, and OSA circumstances is able to increase HIF-1α expression in white blood cells (Serebrovska et al., 2017; Tregub et al., 2020; Fitzpatrick et al., 2021; Nanduri et al., 2021). Thus, both mechanisms of action of hypoxia are active in intermittent regimes, and the direction and modality of effect are dependent on the level of inflammation and interaction of multiple HIF-1α targets.

**FIGURE 2:**
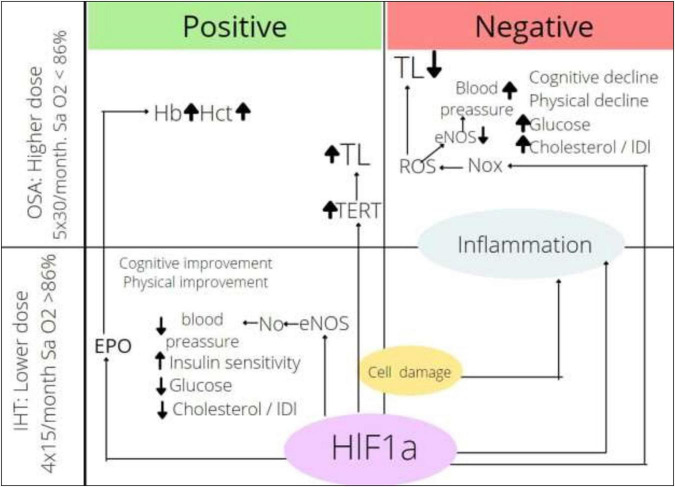
Effects of intermittent hypoxia depending on dose. HIF-1α, hypoxia inducible factor-1α; ROS, reactive oxygen species; Nox, nicotinamide adenine dinucleotide phosphate oxidase; eNOS, endothelial nitric oxide synthase; TERT, telomerase reverse transcriptase; TL, telomere length; EPO, erythropoietin; Hb, hemoglobin content; Hct, hematocrit; IHT, intermittent hypoxic training; OSA, obstructive sleep apnea.

The effects of intermittent hypoxia depending on the dose of hypoxia and the mechanism of actions are presented in Figure 2. Endothelial nitric oxide synthase (eNOS) is activated by HIF-1α; in the regime of IHHT and IHNT, its activity is upregulated, which causes more intensive NO production followed by vassal tonus and blood pressure decrease. At the same time, NOx is also the HIF-1α target. NOx is activated in OSA circumstances, which causes ROS production, NO inactivation, and blood pressure increase. It looks like the effect of NOx stimulation overwhelms the effect of NOS stimulation by the same transcriptional factors under more severe hypoxia. We can also speculate that HIF-1α → EPO → Hb axis is too weak in the training regime, whereas it gains strength under more severe hypoxia. It is very important to find factors that mediate the effect of intermittent hypoxia. HIFs are certainly candidates for the role of mediators, although other groups of molecules cannot be excluded. It is a fact that HIF-1a expression is upregulated in both OSA and IHT; however, it is not well known whether the oxygen-dependent pathway or hypoxic tissue destruction, accumulation of pro-inflammatory molecules, or activation of MAPK-1 pathway is the main factor in HIF-1a accumulation. The role of HIF-1a downstream is not clear either. On the one hand, the activation of glycolysis ensures the adaptation of the whole organism to hypoxic conditions; on the other hand, the accumulation of HIF-1a initiates the Warburg effect in leukocytes, increasing the rate of ATP-dependent processes tenfold. It can be speculated that exacerbation of inflammation in chronic conditions may be one of the mechanisms of the therapeutic effect of intermittent hypoxia. On the other hand, HIF-1a-dependent activation of inflammation can act destructively. It can be assumed that the direction of the effect of intermittent hypoxia depends on the balance between the metabolic and pro-inflammatory effects of HIF-1a.

It should be noted that not very much is known about the effects of intermittent hypoxia on rejuvenation markers. Nevertheless, the dose of hypoxia appears to be a key when an important aging marker, TL, is discussed. Telomeres protect chromosomes from end-to-end fusions and that is why they influence many processes in cellular metabolism. There is a discrepancy in the pull of data dedicated to TL in OSA. The majority of studies point out the decrease of TL and correlation between the severity of OSA or/and age of persons and TL (Boyer et al., 2016). Other authors stated that telomere shortening in OSA is not dependent on syndrome severity, age, or co-morbidity (Barceló et al., 2010). On the contrary, OSA in children causes rather telomere elongation than shortening (Kim et al., 2010). The implications of OSA impact on TL remain to be defined; nevertheless, some speculations can be formulated. The length of telomeres decreases in each cycle of division due to the inability of DNA polymerase to completely replicate the terminal region. Also, telomeres can be damaged independent of the cell cycle directly by oxidative stress. The length of telomeres in different tissues depends on the proliferation of this tissue, but always correlates with age (Vaiserman and Krasnienkov, 2021). The restoration of TL is provided by telomerase reverse transcriptase (TERT). There is a lot of evidence that HIF-1α is involved in the upregulation of TERT expression (Lou et al., 2007; Tsai et al., 2011; Liu et al., 2016; Mahfouz et al., 2017; Lu et al., 2021; Song et al., 2021).

Hypoxia affects TL in at least two ways: (1) It causes telomere shortening due to direct damage by oxidative stress products and (2) it increases the expression and activity of telomerase, which lead to the lengthening of telomeres. The balance between the two processes determines the result. In adults suffering from age-related pathologies, the sensitivity to oxidative stress is high, and severe hypoxic exposure to OSA causes telomere shortening. When children or younger people suffer from OSA, telomere shortening is not pronounced. High altitude hypoxia (5,000 m) causes both tissue damage due to oxidative stress and telomere elongation due to HIF-1α-TERT axis activation (Wang et al., 2018). Physical exercise, which is always accompanied by moderate intermittent hypoxia, increases TL in older persons (Sánchez-González et al., 2021; Sellami et al., 2021); it can be speculated that when intermittent hypoxia is applied in a training regime, oxidative stress is minimal; an increase in telomerase expression would lead to telomeres elongation, thereby increasing rejuvenation.

### Limitations

There are some limitations in our review. First, although we observed the effects of IHNT, IHHT, and OSA on some aging markers, the strength of evidence might be weak due to insufficient data, especially in human studies. Second, variations in the study design include the use of human and cell line models; the human models include the use of different study participants such as healthy individuals or patients with different age-related diseases and different age groups; and variations in biomarkers measured hindered us to pool the data for meta-analysis and may also account for some of the conflicting results.

### Conclusion

Intensive literature search was a necessary step in understanding what IHT (IHNT and IHHT) can really do for rejuvenation and in planning research. IHHT and IHNT show positive effects on age-related diseases including cognitive and physical improvement, increased quality of life, downregulation of glucose and cholesterol/LDL, decreased blood pressure, expression of senescence-associated markers; increase resistance to pro-apoptotic stimuli; prolong periods of osteogenesis; and protect the cells from oxygen–glucose deprivation/re-oxygenation stress. On the contrary, OSA causes hypertension, metabolic syndrome, vascular function impairment, quality of life and cognitive scores reduction, advanced brain aging, and increase in insulin resistance, plasma hydrogen peroxide, GSH, IL-6, hsCRP, leptin, and LTL shortening. Thus, it can be speculated that the main factors that determine the direction of the intermittent hypoxia action are intensity and duration of exposure. There is no direct study to prove that IHNT and IHHT actually increase life expectancy in humans. Therefore, further study is needed to investigate the actual effects of IHNT and IHHT on aging in humans.

## Data Availability Statement

The original data presented in the study are included in the article/Supplementary Material, further inquiries can be directed to the corresponding author.

## Author Contributions

BT, ZS, and EE designed the review. BT searched articles and drafted the manuscript. BT and BK selected relevant articles and extracted the data. ZS, US, BK, and EE reviewed the manuscript. All authors contributed to the article and approved the submitted version.

## Conflict of Interest

EE is a co-owner of CellAir Construction GmbH. The remaining authors declare that the research was conducted in the absence of any commercial or financial relationships that could be construed as a potential conflict of interest.

## Publisher’s Note

All claims expressed in this article are solely those of the authors and do not necessarily represent those of their affiliated organizations, or those of the publisher, the editors and the reviewers. Any product that may be evaluated in this article, or claim that may be made by its manufacturer, is not guaranteed or endorsed by the publisher.
