# Supplementary material for: Effects of Intermittent Hypoxia in Training Regimes and in Obstructive Sleep Apnea on Aging Biomarkers and Age-Related Diseases: A Systematic Review
Source: Front Aging Neurosci. 2022 May 23;14:878278. doi: 10.3389/fnagi.2022.878278 (PMC9168371; doi:10.3389/fnagi.2022.878278)
Supplement: Supplementary file 1 [file Data_Sheet_1.docx]

**Supplementary Table S1:** The effects of intermittent hypoxia-normoxia training (IHNT) on aging markers and age related diseases

| **Author,**  **Year**  **(Ref.)** | **Country** | **Condition** | **Age in years** | **No of cases** | **No of controls** | **Aim of the study** | **Study design** | **Type of hypoxia** | **Hypoxia protocol** | **Protocol for controls** | **Organ /tissue/cells** | **Sample** | **Aging markers** | **Method for measurement** | **Results** | **Conclusions** | **Safety issues** |
| --- | --- | --- | --- | --- | --- | --- | --- | --- | --- | --- | --- | --- | --- | --- | --- | --- | --- |
| Bao et al., 2018 | China | Dizziness | 35-62 | 26 | 26 | To study the effect of IHNT for treating dizziness. | RCT | IHNT | 10% O2 for 50 min , 5 times per week for 4 weeks .Each session has 5 cycles of 10% O2 for 5 min followed by room air for 5 min. | No intervention | NA | NA | Dizziness | DHI, ABC, and VVAS | There were significant differences between IHNT group and control group in DHI, ABC, VVAS scores and attack frequencies of dizziness in the end of 4th week. | IHNT could improve dizziness after intervention in the end of 4th week. IHNT could be the effective method for releasing dizziness. | Safe |
| Costalat et al., 2018 | France | Overweight and obese | mean, 56.2 | 3 | 3 | To investigate the effects of moderate IHNT on key cardio-metabolic risk factors. | RCT | IHNT | 10% O2 repeated cycles with normoxia for 70min, 10 sessions, 5 days per week for two weeks | Sham (21% O2) | NA | Blood | blood glucose and lactate | Cholestech LDX device for blood glucose profiles, hand-held portable analyzer for Lactate | Glucose decreased and Lactate increased following a single IHNT session, but no sustained change after 10 sessions of IHNT occurred | IHNT appears to be a safe and effective non-pharmacological method of reducing key cardiovascular risk factors associated with metabolic disorders. | safe |
|  |  |  |  |  |  |  |  |  |  |  |  |  | HDL and LDL | Cholestech LDX device | LDL, LDL/HDL were all significantly decreased after 10 sessions. |  |  |
|  |  |  |  |  |  |  |  |  |  |  |  |  | SBP and DBP | Controlled finger photoplethysmograph | SBP was significantly decreased after 10 sessions. No significant effects on DBP |  |  |
|  |  |  |  |  |  |  |  |  |  |  |  |  | Cardiac autonomic indices (RMSSD and DFAα1) | Controlled finger photoplethysmograph | No significant effects on heart rate,RMSSD, and DFAα1 |  |  |
| Faulhaber et al., 2015 | Switzerland | COPD | mean, 50 (IHHT), 55 (control) | 8 | 8 | To provide information on heart rate and blood pressure responses to IHNT in COPD patients. | RCT | IHNT | 15%–12% FiO2 for 3 weeks.5 sessions/ week, each consisting of 3 to 5 cycles, each cycle 3–5 min with 3-min breaks between cycles. | Sham hypoxia (normal air). | heart | NA | SBP and DBP, heart rate | Heart rate and SaO2 by pulse oximeter, SBP and DBP by automatic wrist blood pressure monitor | During IHNT, no between-group differences were detected for blood pressure or rate pressure product values. Changes in heart rate were significantly different between groups in the course of the 3 weeks, with post hocdifferences only in week 3. | IHNT resulted in specific and moderate heart rate and blood pressure responses, and did not provoke a progressive increase in blood pressure . | safe |
| Muangritdech et al., 2020 | Thailand | Hypertensive patients | Mean range, 47-51 | 15 (IHR) and 15  (IHT) | 17 | To examine the effects of IHNT at rest (IHR) or during exercise (IHT) on blood pressure, NOx and HIF-1α levels over 6 weeks. | RCT | IHNT | 8 events of FIO2 14%, and FIO2 21% for 6 weeks. Each cycle consisted of 8 cycles × 3-min hypoxic air alternating with 3 min normoxic air). | No intervention | NA | Blood | blood pressure | Automatic blood pressure monitor | a significant decrease of the SBP in both IHR and IHT groups at days 2 and 28 post-intervention, respectively. | IHNT may act as an alternative therapeutic strategy for hypertension patients probably through elevation of NOx and HIF-1α production. | not metnioned |
|  |  |  |  |  |  |  |  |  |  |  |  |  | Plasma NOx | Commercial kits | IHR and IHT had increased NOx. At 2 days post-intervention, NOx was negatively correlated with SBP in IHT. |  |  |
|  |  |  |  |  |  |  |  |  |  |  |  |  | HIF-1α | ELISA | IHR and IHT had increased HIF-1α . At 2 days post-intervention, HIF-1α was negatively correlated with SBP in IHT. |  |  |
|  |  |  |  |  |  |  |  |  |  |  |  |  | MDA levels | Spectrophotometer (Draper et al. 1993 method) | After 6 weeks of IHNT, MDA decreased to similar levels in the IHR and IHT compared to the control. |  |  |
|  |  |  |  |  |  |  |  |  |  |  |  |  | The time to complete a 6-min walk. | The 6‑min walk test (6MWT) | Improved walk distance was maintained at day 28 post-intervention, compared to the control, for both the IHR and IHT. |  |  |
| Schega et al., 2013 | Germany | Healthy | 60-70 | 17 | 17 | To evaluate to the effect of IHNT with physical activity compared to solely strength-endurance training on cognitive performance and QoL. | RCT | IHNT | 3 sessions/ week, one session lasting 1 h, hypoxic air for 10 min and normoxia for 5 min. In the first 2 weeks, SpO 2 90%. In the third week, 85%. and 80% of SpO 2 for the remaining 3 weeks. | placebo air that provoked Spo2 94–98% in the blood. | NA | NA | Cognitive performance | the d2 test and the Number Combination Test(ZVT) | A time × group effect was observed by d2 test. No interaction effect was discovered by ZVT. | IHNT combined with physical exercise augments the positive effects of exercise on cognitive performance and QoL in elderly humans. | not metnioned |
|  |  |  |  |  |  |  |  |  |  |  |  |  | quality of life (QoL) | SF-12 and PSQI) | An interaction effect was not found in the SF-12. But, an interaction effect was observed for sleep quality by PSQI. |  |  |
| Schega et al., 2016 | Germany | Healthy | 60-65 | 18 | 18 | To investigate the effect of IHNT combined with aerobic training on hematological parameters and physical performance as well as peripheral levels of the neurotrophin BDNF and cognitive function. | RCT | IHNT | Hypoxia for 90 min followed by an aerobic training on bicycle ergometers for 30 min under ambient air. 3 times/ week for 4 weeks. In the first week, theSpO2 between 90 and 85% and from the second to the fourth week 80%. | placebo air (SpO2: 95–99%). | NA | NA | Physical performance | spiroergometry and the Stroop test | Increases the time to exhaustion | hypoxic training seems to be beneficial to enhance hematological parameters, physical performance and cognitive function in older people. The current hypoxic-dose was not able to enhance the serum BDNF-level or˙VO2 max. | not metnioned |
|  |  |  |  |  |  |  |  |  |  |  |  |  | cognitive function | spiroergometry and the Stroop test | an augmented and sustainable improvement in cognitive function |  |  |
|  |  |  |  |  |  |  |  |  |  |  |  |  | hematological parameters (RBC, Hgb, Hct) | Standard hematological analyzer | increases in the values of hematological parameters |  |  |
|  |  |  |  |  |  |  |  |  |  |  |  |  | serum BDNF-level. | BDNF DuoSet ELISA kit. | In both groups, the˙VO2 max and serum BDNF-level did not increase |  |  |
| Tobin et al., 2020 | Australia | Healthy | mean, 67 | 50 | 50 | To investigate the effects of intermittent and continuous hypoxia on key markers of haematological adaptation, stress and cardiac damage in healthy senior participants. | Uncontrolled CT | IHNT and CHT | 3 phases of training for 3 weeks: 2 weeks normal air and normoxia,  5 days intermittent (IH) hypoxia (SpO2 of 85% ) for 70 min . After a 5-month washout period, continuous hypoxia (CH) (SpO2= 85%, for 70 min). | 5 days room air without a mask forllowed by 5 days sham (FiO2=21) with mask. | NA | Saliava for S‑IgA and cortisol and Blood for all other markers | RBC count, Hgb Hct | Beckman Coulter® Ac·T diff™ Analyzer | RBC, and Hgb only increased by day 5 of IH treatment compared to day 5 baseline values and day 5 sham values. Hct did not change in either Sham-IH or Sham-CH regardless of the baseline values. | These results revealed that inherent differences in the IH and CH hypoxic patterns could provide crucial components required to trigger hematological changes in senior individuals, without eliciting immunological stress responses or damaging the myocardium. | safe |
|  |  |  |  |  |  |  |  |  |  |  |  |  | percentage of reticulocytes (% Retics), | BD FACSCalibur ™ Flow Cytometry | % Retics did not change in either Sham-IH or Sham- CH regardless of the baseline values . |  |  |
|  |  |  |  |  |  |  |  |  |  |  |  |  | The OFF-score | OFF‑score= [Hb] ⋅ 10 − 60⋅√%Retics | OFF-score value increased only during the final day of IH treatment. |  |  |
|  |  |  |  |  |  |  |  |  |  |  |  |  | S-IgA, cortisol, and cTnT | S‑IgA and cortisol by indirect competitive immunoassay and direct competitive immunoassay,respectively. cTnT by Cobas ® Troponin T STAT kit. | No difference was observed in S-IgA, cortisol or cTnT following IH or CH. |  |  |
| Törpel et al., 2018 | Germany | Healthy | 18-35 (young) and 60-75 (old) | 59 | 64 | To investigate the dose–response relationship regarding the duration of hypoxia until an EPO expression and the amount of EPO expression in old vs. young cohorts. | RCT | IHNT | The intensity of hypoxia was adjusted for 3 h with two approaches: 1) FiO2 13.5% (H-ext), and 2) the FiO2 (H-int) was individually adjusted to an SaO2 of the blood of 80%. | A placebo air (FiO2 21%). | Kidney | Blood (serum) | EPO | two-side sandwich enzyme-enhanced chemiluminescent immunoassay method (CLIA) | EPO increased significantly after 180 min in both cohorts (young and old) and in both investigations (H-ext and H-int ). | After 180 min hypoxia, EPO increases significantly in both age cohorts. The amount of EPO expression is significantly higher in young people during the same internal intensity of hypoxia than in old people. | safe |
| Zhang et al., 2019 | China | Lung cancer | Mean 62 (HPC), 63 (control) | 35 | 35 | To test the effect of hypoxic preconditioning (HPC) before surgery in patients with lung cancer. | RCT | IHNT | FiO2 was initially set at 60%, and in cases of saturation of pulse oxygenation (SpO2) less than 92%, FiO2 was increased to 100%. Three cycles of 5-min hypoxia and 3-min ventilation applied to the nondependent lung served as the HPC intervention. | patients received two-lung ventilation | lung | NA | PaO2/FiO2 ratio, and pulmonary function | pulmonary function of forced vital capacity (FVC) and forced expiratory volume in 1 s (FEV1) by spirometer | HPC significantly increased the PaO2/FiO2 ratio compared with the control at 30 min after one-lung ventilation and 7 days after operation. Compared with the control, it also significantly improved postoperative pulmonary function | HPC improves postoperative oxygenation, enhances the recovery of pulmonary function, and reduces the duration of hospital stay in patients undergoing thoracoscopic lobectomy. |  |
|  |  |  |  |  |  |  |  |  |  |  |  |  | postoperative pulmonary complications | Postoperative pulmonary complications (pneumonia, atelectasis, pleural effusions, and prolonged air leak > 7 days). | No significant differences between groups were observed in the incidence of pulmonary complications or overall postoperative morbidity. |  |  |
|  |  |  |  |  |  |  |  |  |  |  |  |  | duration of hospital stay | The duration of hospital stay was counted from the day of operation to the day of discharge. | Markedly reduced the postoperative hospital stay duration. |  |  |

IHNT= Intermittent hypoxia-normoxia training; IH=Intermittent hypoxia; RCT= Randomized controlled clinical trial; DHI=The Dizziness Handicap Inventory; ABC= Activities-specific Balance Confidence Scale; VVAS= Vertigo Visual Analog Scale; HDL= High- density lipoprotein; LDL= low-density lipoproteins; SBP=systolic blood pressure; DBP= diastolic blood pressure; RMSSD=[root mean square of successive R-R interval differences; DFAα1=short-term fractal scaling exponent; COPD= chronic obstructive pulmonary diseases; SaO2= O2 saturation; NOx =nitric oxide metabolites; HIF-1α= Hypoxia-inducible factor-1alpha; MDA= Malondialdehyde: QoL= Quality of life; SF-12=The Medical Outcomes Study Short-Form 36-Item Health Survey; PSQI= Pittsburgh Sleep Quality Index; BDNF=brain-derived eurotrophic factor; S-IgA= secretory immunoglobulin A; RBC= red blood cells; Hgb=hemoglobin; Hct= hematocrit; cTnT= cardiac troponin T; EPO=Erythropoietin; CHT= Continuous hypoxia training

**Supplementary Table S2:** The effects of intermittent hypoxia-hyperoxia training (IHHT) on aging markers and age related diseases

| **Author,**  **Year**  **(Ref.)** | **Country** | **Condition** | **Age in years** | **No of cases** | **No of controls** | **Aim of the study** | **Study design** | **Type of hypoxia** | **Hypoxia protocol** | **Protocol for controls** | **Organ /tissue/cells** | **Sample** | **Aging markers** | **Method for measurement** | **Results** | **Conclusions** | **Safety**  **issues** |
| --- | --- | --- | --- | --- | --- | --- | --- | --- | --- | --- | --- | --- | --- | --- | --- | --- | --- |
| Dudnik et al., 2018 | Russia | Cardiac patients with comorbidities | Mean 66 (IHHT), 65 (control) | 15 | 14 | To compare IHHT to a standard exercise-based rehabilitation program with respect to cardiorespiratory fitness (CRF) in older cardiac outpatients. | RCT | IHHT | 15 sessions hypoxia (11–12%O2)—hyperoxia (30–33%O2) for 5 weeks: 3 sessions/ week, 5–7 hypoxic periods of 4–6 minutes, 3minute hyperoxic recovery. | 15 sessions sham hypoxia exposure (room air), and 8-week exercise program | heart | blood | CRF | Indirect calorimetry (Bruce and M-Bruce protocols). | CRF in the IHHT group was not significantly different compared with the control group. Systolic and diastolic blood pressures were not significantly different between groups after treatment. | IHHT might be a suitable option for older patients who cannot exercise. A 5-week IHHT is as effective as an 8-week exercise program in improving CRF, without hematological changes. | Safe |
|  |  |  |  |  |  |  |  |  |  |  |  |  | Hematological markers (Hgb, RBC, reticulocyte) | Blood biochemistry analyzers | Hgb content was not significantly different between groups. RBC and reticulocytes did not change pre/post interventions in both experimental groups. |  |  |
| Glazachev et al.,2017 | Russia | coronary artery disease (CAD) | 43-83 | 27 | 19 | To investigate the effects of IHHT on exercise tolerance, cardiometabolic risk factors, and patient-relevant subjective parameters in CAD patients. | Non RCT | IHHT | 15 sessions hypoxia (10% O2)- hyperoxia (30%O2). 3 sessions / week, 5 to 7 hypoxic periods lasting 4 to 6 minutes, with 3-min hyperoxic recovery. | O2 at 21% | heart | blood | Exercise performance | Anthropometrics, resting blood pressure and heart rate tests, cardiopulmonary stress test, Echocardiographic study in M-mode. | The IHHT showed improved exercise capacity, reduced systolic and diastolic blood pressures, enhanced left ventricle ejection fraction, but only at 1-month follow-up. | IHHT is associated with improved exercise tolerance, healthier risks factors profile, and a better quality of life. The study also suggests that IHHT is as effective as an 8-week standard rehabilitation program in CAD patients. | safe |
|  |  |  |  |  |  |  |  |  |  |  |  |  | Blood markers (RBC, Hgb, reticulocyte), and metabolic profiles (serum total, LDL and HDL,triglycerides, and glucose) | Analyzed using standardized analytical methods. | Hgb and glycemia were unchanged after IHHT, but glycemia was significantly lower at the 1-month follow-up. Total cholesterol and LDL were lower after IHHT. At the 1-month follow-up total cholesterol was similar to pretreatment. Reticulocytes were significantly higher in the IHHT at the end of treatment and at 1-month follow-up. |  |  |
|  |  |  |  |  |  |  |  |  |  |  |  |  | Quality of life | Seattle Angina Questionnaire [SAQ]. | The SAQ profile was improved and not significantly different to the control after standard rehabilitation.  The IHHT was compared to the control at 1-month follow-up, and no differences were found. |  |  |
| Serebrovska et al., 2019 | Ukraine | Prediabetic patients | 51-74 | 17  (IHHT), 22 (IHNT) | 16 | To compare the impact of adaptation to IHHT versus IHNT on some metabolic variables in prediabetic patients. | RCT | IHHT and IHNT | 15 sessions IHHT and IHNT, 5 times/ week for 3 weeks. Each session consisted of 4 cycles of 5 min of 12%FiO2 followed by 3 min of 33% O2 in nitrogen or 5 minutes of normoxia | IHHT placebo without switching to the supply of hypoxic gas mixture. | NA | Blood (Serum and plasma) | Serum total cholesterol, HDL, LDL, and triglycerides | Cholestech LDXdevice | The study showed the same positive effect of IHNT and IHHT: decreased total blood cholesterol and LDL; and an equally smaller drop in SpO2 during acute hypoxic test. Improved parameters persisted 1 month after training termination in both groups. | One of the advantages of IHHT over IHT observed in this study could be some reduction in the duration of the sessions due to shortening reoxygenation periods. | Not mentioned |
|  |  |  |  |  |  |  |  |  |  |  |  |  | Plasma glucose concentrations | glucose oxidase method on a semiautomatic biochemical analyzer | The study showed the same positive effect of IHHT and IHNT: equal reduction of serum glucose concentrations, both fasting and 2 hours of OGTT. Improved parameters persisted 1 month after training termination in both groups. |  |  |
| Susta et al., 2020 | Russia | Healthy | 18-24 | 21 | Cobared with baseline | To measure oxidative stress and antioxidant capacity in healthy humans after being acutely exposed to both IHN and IHH. | Uncontrolled CT | IHHT and IHNT | FIO2 11% for up to 7 min followed by 3–5 min of exposure to normoxia (room air) or hyperoxia, FIO2 30%-35% | before exposure to IHHT or IHNT (baseline results) | NA | Blood (plasma) | oxidative stress(concentration of hydroperoxides) | The d-ROMs fast test | Oxidative stress was similar after IHN and IHH exposures compared with baseline values. | hypoxia (IHN and IHH) cause neither pronounced oxidative stress nor antioxidant capacity impairment in healthy humans. | safe |
|  |  |  |  |  |  |  |  |  |  |  |  |  | antioxidant capacity | The PAT test | The antioxidant capacity was also similar between experimental groups after both modalities of exposure. |  |  |

IHHT= Intermittent hypoxia-hyperoxia; IHNT= Intermittent hypoxia-normoxia training; RCT= Randomized controlled clinical trial; CRF= cardiorespiratory fitness; RBC= red blood cells; Hgb=hemoglobin; Hct= hematocrit; SAQ=Seattle Angina Questionnaire

**Supplementary Table S3:** The effects of continuous hypoxia training and intermittent hypoxia-normoxia training (IHNT) on aging markers and age related diseases using cell culture model

| **Author, year (Ref.)** | **Country** | **Targeted conditions** | **Cell line** | **Aim of the study** | **Study design** | **Type of hypoxia** | **Hypoxia protocol (for cases)** | **Protocol (controls)** | **Aging markers** | **Method for measurement** | **Results** | **Conclusions** | **Safety issues** |
| --- | --- | --- | --- | --- | --- | --- | --- | --- | --- | --- | --- | --- | --- |
| Casciaro et al., 2020 | Italy | Cellular aging | hAFSCs | To evaluate various aging features of hAFSCs cultured at different oxygen concentrations | non RCT | CHT | 1% oxygen for 5 weeks upto 8-9 passages. | 20% oxygen | Stemness properties (mRNA levels of Oct4 upregulation and protein expression of SSEA4) | mRNA levels of Oct4 by RT- PCR, protein expression of SSEA4 by SDS PAGE and western blot | 1% O2 extends stemness | Low oxygen concentrations might improve the generation of functional hAFSCs for therapeutic use by delaying the onset of cellular aging. | Not reported |
|  |  |  |  |  |  |  |  |  | Proliferative ability | EdU method | 1% O2 extends proliferative features |  |  |
|  |  |  |  |  |  |  |  |  | Induction of senescence-associated markers | processed as reported by Dimri et al. 1995 | 1% O2 delays induction of senescence-associated markers. |  |  |
|  |  |  |  |  |  |  |  |  | Changes in metabolism and resistance to stress | SDS PAGE and western blot | Hypoxic hAFSCs activate a metabolic shift and increase resistance to pro-apoptotic stimuli. |  |  |
|  |  |  |  |  |  |  |  |  | Osteogenic differentiation | Differentiation assays | cells at low oxygen remain capable of osteogenesis for prolonged periods of time |  |  |
| Damiani et al., 2018 | Italy | Skin aging | HDF | To examine the influence of oxygen tension on cell aging in HDF and how this impacted ROS production, the enzymatic and non-enzymatic antioxidant response system, and the efficacy of this defense system in limiting DNA damage and in modulating gene expression of proteins involved in the extracellular matrix, linked to skin aging. | non RCT | CHT | 5% O2. HDF were passaged at 80% confluence. Cells were serially cultured until enough cells were obtained for all experiments. | 21% O2 | cellular proliferation rate | Flow cytometry | increased cell proliferation under 21% O2 compared to 5% O2 | The 21% O2 impose a mild oxidative stress on dermal fibroblasts which accelerates the aging process in culture compared to 5% O2 where the underlying level of oxidative stress is reduced. cells grown under normoxia undergo a “stress-induced premature senescence” when compared to their matched counterparts grown under hypoxia.  The modulation of miR-181a to different oxygen tensions and its potential role in altering the expression of antioxidant genes could represent an important molecular event in skin aging that may be addressed as a target for antiaging strategies. | not reported |
|  |  |  |  |  |  |  |  |  | Intracellular ROS | The leuco dye carboxy-2,7-dichlorofluorescein diacetate (carboxy-H2DCFDA) | Lower levels of intracellular ROS in cells at 21% O2 compared to those at 5% O2. |  |  |
|  |  |  |  |  |  |  |  |  | mitochondrial superoxide anion generation | FlowCellect™ MitoStress Kit | Higher levels of mitochondrial superoxide anion in cells at 21% O2 compared to at 5% O2 |  |  |
|  |  |  |  |  |  |  |  |  | CoQ10 level and oxidative status | HPLC | Total Coenzyme Q10 levels decrease with cell passages and increase with oxygen tension. |  |  |
|  |  |  |  |  |  |  |  |  | Total glutathione (GSH + GSSG) | spectrophotometry | Total Glutathione levels reduce under Low oxygen tension |  |  |
|  |  |  |  |  |  |  |  |  | single and double-strand DNA damage | Comet assay | DNA damage increased under 21% O2 vs 5% O2. |  |  |
|  |  |  |  |  |  |  |  |  | β-galactosidase activity, p16, CAT, SOD1, SOD3, MMP1, and COL1A1 genes expression | qPCR | higher levels of SOD1 and SOD3, upregulation of MMP1 and downregulation of COL1A1 under 21% O2 vs 5% O2. |  |  |
| Minamino et al., 2001 | USA | Vascular disorder | VSMC | To demonstrate the causal role of telomerase activation and telomere function for the long-term growth and viability of VSMC under conditions of hypoxia. | non RCT | CHT | 1% O2 until 10 passages. | 21 % O2 | telomerase activity | stretch PCR and modified telomeric repeat amplification assays | Chronic hypoxia can prolong the growth of human VSMC by inducing telomerase activity and telomere stabilization. Hypoxia induced phosphorylation of the telomerase catalytic component (TERT) and sustained high levels of TERT protein expression in VSMC compared to normoxia. | Hypoxic induction of telomerase activity could be involved in long-term growth of VSMC and may thus contribute to human vascular disorders. | Not reported |
| Tantingco et al., 2020 | USA | Ischemic stroke | mice microglia, EOC20 cells | To examine the role of intermittent hypoxic training in the regulation of microglia polarization that occurs in the in vitro model of oxygen– glucose deprivation –reoxygenation. | non RCT | IHNT | Three days IHT consisting of five to eight daily, 5- to 10-min cycles of hypoxia (4–3.5% O2) with intervening 4-min reoxygenation. | 21 % O2 | cell viability | Calcein AM and LDH activity assay | Intermittent hypoxic training protects the microglia from oxygen–glucose deprivation /reoxygenation stress. | Due to the effect of intermittent hypoxic training on the microglia phenotype, intermittent hypoxic training could be considered as an effective intervention in the treatment or rehabilitation program for the ischemic stroke victims. | Not reported |
|  |  |  |  |  |  |  |  |  | TLR2 proteins content | Western blot | The TLR2 protein content was significantly elevated in the oxygen–glucose deprivation and reoxygenation group, and intermittent hypoxic training lowered it to normoxia level. |  |  |
|  |  |  |  |  |  |  |  |  | anti-inflammatory cytokines (IL-10 and IL-4) | ELISA | IL-10 and IL-4 were significantly increased in the intermittent hypoxic training groups. |  |  |
|  |  |  |  |  |  |  |  |  | reactive oxygen species (ROS) | H2DCFDA assays | intermittent hypoxic training lowers the ROS generation |  |  |
|  |  |  |  |  |  |  |  |  | phagocytic activity | latex beads coated with FITC | Intermittent hypoxic training increases phagocytic activity (about 12 folds) vs. normoxia. |  |  |
|  |  |  |  |  |  |  |  |  | cell phenotype | Immunocytochemistry, flow cytometric analysis, and immunoblot CD206 (M2). | Intermittent hypoxic training regulates the polarization of the microglial phenotype toward anti-inflammatory type M2. |  |  |
| Polonis et al, 2020 | USA | OSA | HWPs | To examine the effects of IH, a hallmark of OSA, on senescence in human white preadipocytes. | non RCT | IHNT | 9 cycles of IH (30 min of 21% O2 followed by 30 min of 0.1% O2) per day for up to 7 consecutive days | 21%O2 | senescence in HWPs | Senescence associated β-galactosidase staining | A higher prevalence of cells positive for senescence-associated β-galactosidase activity was also evident with chronic IH exposure. | This study identifies chronic IH as a trigger of senescence-like phenotype in preadipocytes. | Not reported |

hAFSCs = Human amniotic fluid stem cells; CHT= continuous hypoxia training; IHT= Intermittent hypoxia training; IH= intermittent hypoxia; non RCT=non randomized controlled trial; HDF= human dermal fibroblasts; VSMC=vascular smooth muscle cell; HPLC= high performance liquid chromatography; qPCR=quantitative polymerase chain reaction; ELISA= enzyme linked immunosorbent assay; OSA = Obstructive sleep apnea; HWPs = human white preadipocytes; ROS= reactive oxygen species; TLR2= toll-like receptor 2

**Supplementary Table S4:** The effects of sleep apnea on aging markers and age related diseases

| **Author, Year (Ref.)** | **Country** | **Condition** | **Age in years** | **No of cases with SA** | **No of controls** | **Aim of the study** | **Study design** | **Total quality score^#^** | **Type of sleep apnea** | **Sleep apnea criteria** | **Criteria for controls** | **Organ /tissue/cells** | **Sample** | **Aging markers** | **Method for measurement** | **Results** | **Conclusions** |
| --- | --- | --- | --- | --- | --- | --- | --- | --- | --- | --- | --- | --- | --- | --- | --- | --- | --- |
| Addison-brown et al., 2014 | USA | SA | 47–93 | 782 | 2143 | To assess the associations of OSA with cognition and quality of life and whether these vary with age. | case-control | 8 | OSA | High risk of OSA based on the Berlin Sleep Questionnaire. | low risk of OSA | NA | NA | Cognitive function | Standardized fluency and recall measures. | Those at high risk for OSA had significantly lower cognitive scores. However, some of the associations were age-dependent. Differences in cognition between those at high and low OSA risk were most pronounced during middle age, with attenuated effects after age 70 years. | Authors able to confirm OSA’s effect on cognition, depressive symptoms and HRQoL. They also found differential effects based on age, with more detrimental correlates of OSA in younger versus older adults, particularly in terms of mood and HRQoL. |
|  |  |  |  |  |  |  |  |  |  |  |  |  |  | Depression | Center for Epidemiologic Studies Depression Scale. | Those at high risk for OSA had significantly higher depressive symptoms |  |
|  |  |  |  |  |  |  |  |  |  |  |  |  |  | HRQoL | The Medical Outcomes Study Short Form-12 (SF-12). | Those at high risk for OSA had significantly lower HRQoL. However, some of the associations were age-dependent. Differences in quality of life between those at high and low OSA risk were most pronounced during middle age, with attenuated effects after age 70 years. |  |
| Ayalon et al., 2010 | USA | SA | 25-59 | 14 | 14 | To examine whether the combination of increasing age and sleep apnea will lead to a stronger compensatory response than either factor alone, or overwhelm the brain’s capacity to compensate | case-control | 6 | OSA | Young (<45 yrs) and middle aged (> 45 yrs) sleep apnea patients. Inclusion criteria: apnea–hypopnea index 10 or greater. | Age-matched healthy subjects. AHI <5 | Brain | NA | Cognitive performance and brain activation | fMRI | Middle-aged patients with OSA showed reduced performance for immediate word recall and slower reaction time during sustained attention. For both tasks, decreased activation was detected for middle-aged sleep apnea relative to the other groups in task-related brain regions. | The presence of both sleep apnea and increasing age overwhelmed the brain’s capacity to respond to cognitive challenges with compensatory recruitment and to maintain performance. |
| Duffy et al., 2016 | Australia | SA, and at risk for dementia | Mean, 65 | 24 | Correlation study | To examine the relationship between GSH concentration in the anterior cingulate cortex (ACC) and measures of hypoxemia (O2 desaturation and AHI) in older adults at-risk for dementia. | Cross-sectional | 7 | OSA | SA was determined based on O2-desaturation and AHI, rapid eye movement sleep, and non-rapid eye movement sleep. | Correlation study | Brain | ACC | GSH and creatine | 1H-MRS | Increased levels of GSH/Cr were associated with lower oxygen desaturation and more severe apnea-hypopnea index scores during rapid eye movement sleep. In addition, ACC GSH/Cr correlated with poorer executive functioning (i.e., response inhibition and set shifting). | Markers of nocturnal hypoxemia and sleep disordered breathing (SDB) are associated with cerebral oxidative stress in older people at-risk for dementia, suggesting a potential mechanism by which SDB may contribute to brain degeneration, cognitive decline, and dementia. |
| Edwards et al., 2014 | USA | SA | 20-40 (young) and >60 (old) | 10 young, and 10 old | age groups comparative study | To investigate the effect of aging on four key traits responsible for OSA in a group of young and older patients with OSA. | case-control | 7 | OSA | OSA patients with AHI > 10 events/h and were treated with CPAP for more than 5 h per night for at least 2 mo prior to enrollment. | Age groups comparative study | Upper respiratory system | NA | Pharyngeal anatomy/collapsibility | It was quantified as the ventilation at CPAP = 0 cmH2O immediately after the CPAP drop. | In comparison with younger patients with OSA, older patients had a more collapsible airway | The data suggest that airway anatomy/collapsibility plays a relatively greater pathogenic role in older adults, whereas a sensitive ventilatory control system is a more prominent trait in younger adults with OSA. |
|  |  |  |  |  |  |  |  |  |  |  |  |  |  | loop gain (LG) | the ratio of the ventilatory overshoot to the preceding reduction in ventilation. | In comparison with younger patients with OSA, older patients had a lower LG |  |
|  |  |  |  |  |  |  |  |  |  |  |  |  |  | upper airway muscle responsiveness/gain (UAG) | the ratio of the increase in ventilation to the increase in ventilatory drive across the pressure drop. | It was similar between groups. |  |
|  |  |  |  |  |  |  |  |  |  |  |  |  |  | respiratory arousal threshold | estimated as the ventilatory drive that caused arousal. | It was similar between groups. |  |
| Khan et al., 2011 | USA | SA | >65 | 101 | No non SA control | To identify OSA and anemia of aging (AOA) coexistance, and OSA treatment -AOA interaction. | cohort | 9 | OSA | OSA was diagnosed when AHI was > 5 events/h. | Correlation study | NA | blood | Hct and Hgb | not mentioned | Hct changed significantly post OSA treatment. The change in Hgb after OSA treatment was not significant. However, the change in Hgb was large enough to reach WHO standards for AoA. Hct changed significantly among both men and women. | There was no AOA before OSA treatment. But there was AOA 1 year after OSA treatment. AOA and OSA share common inflammatory processes. Authors believe OSA inflammatory processes interact with OSA hypoxia-induced erythropoiesis. |
| Kritikou et al., 2014 | USA | SA | Mean 42- 66 | 38 | 39 | To examine the association of OSA with sleepiness and inflammation/insulin resistance in a relatively non obese population. | case-control | 8 | OSA | AHI >10 events/h of sleep for females and >15 events/h for males. | Individuals  Without SA | NA | blood (plasma) | Sleepiness | ESS, MSLT and PVT | Apnoeic males were significantly sleepier than controls. CPAP improved subjective sleepiness. | OSA is associated with sleepiness, inflammation and insulin resistance, even in nonobese males, and this association is stronger in males than in females. Short-term CPAP does not improve the inflammatory/metabolic aberrations in OSA. |
|  |  |  |  |  |  |  |  |  |  |  |  |  |  | IL-6 | ELISA | Apnoeic males had significantly IL-6 than controls. Apnoeic females had IL-6 similar to controls. CPAP did not change IL-6 |  |
|  |  |  |  |  |  |  |  |  |  |  |  |  |  | TNFR-1 | ELISA | no significant difference was observed in TNFR-1 values. CPAP did not change TNFR-1 |  |
|  |  |  |  |  |  |  |  |  |  |  |  |  |  | leptin and adiponectin | radioimmunoassay | Apnoeic males had significantly higher leptin than controls. Apnoeic females had leptin and adiponectin similar to controls. CPAP did not change leptin and adiponectin |  |
|  |  |  |  |  |  |  |  |  |  |  |  |  |  | hsCRP | ELISA | Apnoeic males and females had significantly higher hsCRP. CPAP did not change hsCRP |  |
|  |  |  |  |  |  |  |  |  |  |  |  |  |  | fasting glucose and insulin levels | double antibody method using reagents obtained from Millipore Co. | Apnoeic males had significantly higher insulin resistance than controls. Apnoeic females had insulin resistance similar to controls. CPAP did not change insulin resistance |  |
| Sajkov et al., 1998 | Australia | SA and tetraplegia | 19-60 | 11 | 26 | To study the influence of SA on neuropsychological function in tetraplegic patients. | case-control | 7 | OSA | AHI >15 per hour of sleep. | Normal (without  tetraplegic) | NA | NA | Neuropsychological function (eg, memory, perception, attention and concentration) | Rey Auditory Verbal Learning Test, and Digit Span test. | The neuropsychological functions most affected by nocturnal desaturation were: verbal attention and concentration, immediate and short-term memory, cognitive flexibility, internal scanning and working memory. | Sleep apnoea impairs daytime cognitive function in tetraplegia patients, particularly attention, concentration, memory and learning skills. Cognitive disturbances resulting from SA might adversely affect rehabilitation in patients with tetraplegia. |
| Vgontzas et al., 2019 | USA | SA | 20-80 | 103 | 641 | To examine the association between mild and moderate OSA and incident hypertension . | cohort study | 9 | OSA | Mild OSA = AHI 5 to 14.9 events/h, and moderate OSA =AHI 15 to 29.9 events/ h. All cases were without hypertension at the baseline | Normal control = AHI < 5. | NA | NA | Incident hypertension | Incident hypertension was defined by a self-report of receiving antihypertensive medication and/or history of a diagnosis since their baseline study. | Mild-to-moderate OSA was significantly associated with increased risk of incident hypertension. Importantly, this association was modified by age; while strong in young and middle-aged adults, the association was lost in adults older than 60 years . Furthermore, the association of mild-to-moderate OSA with components of metabolic syndrome was strongest in young and middle-aged adults. | Mild-to-moderate OSA, even when asymptomatic, is associated with increased risk of incident hypertension, but the strength of association significantly decreases with age. |
| Weihs et al., 2020 | Germany | SA | Mean 52.5 | 284 (AHI >5) and 218 (ODI>5) | 406 (AHI),472 (ODI) | To determine the association between OSA and advanced brain ageing | cross-sectional | 7 | OSA | Mild to severe OSA (AHI and ODI ≥ 5) and severe OSA (AHI and ODI ≥ 15). | Association study | Brain | NA | A score quantifying age-related brain patterns in 169 brain regions | magnetic resonance imaging | AHI and ODI were both positively associated with brain age. The effects remained stable in the presence of various confounders such as diabetes and were partially mediated by the white blood cell count, indicating a subclinical inflammation process. | The study reveals an association between OSA and brain age, indicating subtle but widespread age-related changes in regional brain structures, warranting further examination of OSA in the prevention of neurodegenerative diseases. |
| Yim-Yeh et al., 2010 | USA | SA | 18-70 | 38 | 34 | To test the hypotesis that OSA, independent of obesity, contributes to abnormal vascular function. | cross-sectional | 6 | OSA | OSA was defined as AHI > 10/h. | Without OSA | NA | NA | Vascular function , and Arterial stiffness | Vascular function was assessed with brachial artery ultrasound, skin microcirculation by laser Doppler flowmetry. Arterial stiffness by arterial tonometry. | FMD was impaired in patients with OSA. OSA did not significantly influence vascular function in the skin microcirculation. The augmentation index, a measure of arterial stiffness, was similar between the OSA and control groups, OSA independently predicted the augmentation index in men only. | In obesity, both OSA and aging impair endothelial function and increase arterial stiffness. The influence of OSA on vascular function is most pronounced in young subjects. OSA, therefore, may be associated with functional impairment (“a premature aging effect”) on the endothelium and on arterial stiffness (in men), although skin microcirculatory function appears preserved. |

# =Total quality score out of 9 for cohort/case-control and out of 7 for cross-sectional; SA= sleep apnea; OSA= obstructive sleep apnea; HRQoL= health related quality of life; AHI= apnea–hypopnea index; fMRI=Functional magnetic resonance imaging; CPAP =continuous positive airway pressure; ESS=Epworth Sleepiness Scale; MSLT= Multiple Sleep Latency Test; PVT=the Psychomotor Vigilance Test; IL-6=interleukin-6; TNFR-1=tumour necrosis factor receptor -1; hsCRP= high-sensitivity C-reactive protein; ODI=Oxygen desaturation index; FMD=flow-mediated dilation; 1H-MRS = Proton Magnetic Resonance Spectroscopy; GSH=Glutathione

**Supplementary Table S5:** The effects of sleep apnea on aging markers (Leukocyte Telomere length) and age related diseases

| **Author,**  **Year**  **(Ref.)** | **Country** | **Conditions** | **Age in years** | **No of cases with SA** | **No of controls** | **Aim of the study** | **Study design** | **Total quality score^#^** | **Type of sleep apnea** | **Sleep apnea criteria (cases)** | **Criteria (controls)** | **Organ /tissue/cells** | **Sample** | **Aging markers** | **Method for measurement** | **Results** | **Conclusions** |
| --- | --- | --- | --- | --- | --- | --- | --- | --- | --- | --- | --- | --- | --- | --- | --- | --- | --- |
| Barcelo et al., 2010 | Spain | SA | 49.5 | 256 | 148 | To compare the TL in OSAS patients with controls and to investigate the relationship of TL to the severity of OSAS, the presence of metabolic  and cardiovascular risk factors. | case-control | 7 | OSAS | AHI > 10 | AHI <10 | Leukocyte | blood | LTL | qPCR | TL was significantly shorter in patients with OSAS than in controls. This difference persisted after adjustment for age, presence of cardiovascular and metabolic changes. TL was not related to the severity of OSAS | TL in circulating leukocytes is shorter in patients with OSAS than controls. The mechanism of this observation is unresolved since it appears independent of chronological age, the severity of OSAS and/or the presence of cardiovascular or metabolic changes. |
| Carroll et al., 2019 | USA | SA | 44 -84 | 322 | 460 | To evaluate whether objectively assessed sleep and sleep apnea relate to LTL | cohort | 9 | OSA | AHI >15 | AHI<15 | Leukocytes | blood | LTL | qPCR | Severe obstructive sleep apnea was associated with shorter LTL. An exploratory analysis found that higher arousal index at Exam 5 was associated with greater LTL decline over the prior 10 years. | OSA was associated with shorter leukocyte telomere length. Individuals with high arousal frequency had greater leukocyte telomere attrition over the prior decade. These findings suggest that sleep apnea and sleep fragmentation are associated with accelerated biological aging. |
| Boyer et al., 2016 | France | SA | 46.8 | 122 | 39 | To determine whether OSAS was associated with telomere shortening independently from the comorbidities associated with this syndrome. | case-control | 6 | OSAS | AHI >5/h | no OSA, AHI ,<5/h | Leukocyte | blood | LTL | qPCR | AHI and oxygen desaturation index were significantly related to telomere shortening. | Intermittent hypoxemia due to OSAS is a major contributor to telomere shortening in middle-aged men. Oxidative stress may explain this finding. |
| Kim et al., 2010 | USA | SA | 5-10 | 111 | 102 | To assess the association between LTL and OSA | case-control | 8 | OSA | AHI> 1/hrTST | children did not snore and had an AHI <1/hrTST | Leukocyte | whole blood | LTL | qPCR | LTL was independently associated with AHI | In pediatric OSA, LTL is longer rather than shorter. Children with OSA have reduced plasma catestatin levels and increased BP along with increased MRP 8/14 levels that exhibit AHI dependencies. Thus, catestatin and MRP 8/14 levels may serve as biomarkers for cardiovascular risk in the context of pediatric OSA. However, the implications of increased LTL in children with OSA remain to be defined. |
|  |  |  |  |  |  |  |  |  |  |  |  |  | Plasma | Catestatin | ELISA | Children with OSA exhibited lower plasma catestatin |  |
|  |  |  |  |  |  |  |  |  |  |  |  |  | Plasma | MRP 8/14 | ELISA | Children with OSA exhibited higher MRP 8/14 levels than controls |  |
| Kim et al., 2016 | Korea | SA | 45.6 | 43 | 34 | To search the predictive markers which reflect the burden of systemic oxidative stress in patients with OSA and whether excessive TL shortening is a characteristic feature that can assess oxidative stress levels. | case-control | 7 | OSA | RDI >5 | RDI<5 | Leukocyte | whole blood for TL, and Plasma for ROS | LTL | qPCR | Significantly shortened telomere length was observed in the circulating leukocytes of the peripheral blood of OSA patients, and telomere length shortening was aggravated more acutely in an age- and BMI-dependent manner. | The results provided evidence that telomere length shortening or excessive cellular aging might be distinctive in circulating leukocyte of OSA patients and may be an predictive biomarker for reflect the burden of oxidative stress in the peripheral blood of OSA patients. |
|  |  |  |  |  |  |  |  |  |  |  |  |  |  | ROS | ELISA | Plasma concentration of hydrogen peroxide was considerably higher in OSA patients, and that this was closely related with the severity of OSA. An inverse correlation was observed between the concentration of hydrogen peroxide and the telomere length of OSA patients and excessive telomere length shortening was linked to severity of OSA. |  |
| Kwon et al., 2015 | Korea | SA | 58.9 yrs | 381 | NA | to examine the objective association between sleep stability and leukocyte telomere lengths | Cohort | 9 | OSA | AHI ≥ 5 | AHI < 5 | Leukocyte | blood | LTL | qPCR | Sleep stability significantly reduced with shortened LTL in OSA patients. | The present study suggested that shorter LTL might contribute to reduced sleep stability by interacting with OSA severity due to the stress of chronic sleep fragmentation or invariant ympathetic activity by respiratory chemoreflex activation. |
| Polens et al.,2019 | USA | SA | 35.6 (cases) and 47.3 (controls) | 73 | 88 | To investigated the association between TL and risk of MACE and cancer in OSA patients. | case-control | 8 | OSA | AHI >5 | AHI < 5 | Leukocyte | blood | LTL | qPCR | The OSA group had a higher likelihood of cancer but no clear evidence of an elevated incidence of MACE compared to the non-OSA group. There was no association between TL and MACE or cancer-risk. | Our study warrants further investigation of any modulating efect of OSA on TL and the risk of MACE and cancer. |
| Polonis et al., 2017 | USA | SA | 27-57 | 106 | 104 | To assess the association beteween OSA and telomere length | case-control | 7 | OSA | AHI ≥ 5 | AHI <5 | Leukocyte | blood | LTL | qPCR | There was no difference in telomere length between OSA and control group. The mean TL in moderate-to-severe OSA was significantly longer than in control group after adjustment for age, sex, BMI, hypertension, dyslipidemia and depression. | Moderate-to-severe OSA is associated with telomere lengthening. These findings support the idea that changes in TL are not unidirectional processes such that telomere shortening occurs with age and disease, but may be prolonged in moderate-to-severe OSA. |
| Riestra et al., 2017 | USA | SA | 30-55 | 35 | 87 | To explore the associations between the risk of OSA and telomere length in African Americans. | case-control | 9 | OSA | High risk of having OSA if scores were positive for two or more of the three categories by Berlin questionnaire. | Patients who scored positively on less than two categories were identified as low risk of having OSA. | Leukocyte | blood | LTL | qPCR | We observed that LTL varied by OSA risk in women. Multiple linear regression analysis confirmed that women at higher risk for OSA presented shorter LTL compared to those at lower risk. These differences were not observed in men. | These findings suggest that OSA risk may contribute to the acceleration of cellular aging processes through telomere shortening. |
| Tempaku et al., 2016 | Brasil | SA | 20-80 | 315 | 613 | To compare the LTL between OSAS patients and controls, as well as to verify the correlation between LTL and sleep parameters. | case-control | 8 | OSAS | AHI > 5 | AHI < 5 | Leukocyte | blood | LTL | qPCR | LTL was significantly shorter in OSAS patients compared to controls. | The study indicates the presence of an association between LTL and OSAS and a significant impact of severity of OSAS in telomeres shortening. |

# =Total quality score out of 9 for cohort/case-control and out of 7 for cross-sectional; SA= sleep apnea; OSA= obstructive sleep apnea; TL=Telomere length; LTL=Leukocyte telomere length; AHI= apnea–hypopnea index; RDI= respiratory disturbance index; qPCR=quantitative polymerase chain reaction; hrTST= hour of total sleep time; MRP= myeloid-related protein; ROS= reactive oxygen species; MACE= major adverse cardiac events.
